# Role of O-specific antigen expression in *Pseudomonas aeruginosa* pathogenicity

**DOI:** 10.1128/mbio.01551-26

**Published:** 2026-10-05

**Authors:** Oliver Hartmann, Matthias Preusse, Ann Kathrin Heroven, Sebastian Felgner, Melisa Gür, Adrian Kordes, Victor Wolff Bengtsen, Mohammad Roghanian, Xinxin Zhao, Mathias Müsken, Nicolas Oswaldo Gomez, Susanne Häussler

**Affiliations:** 1 Department of Molecular Bacteriology, Helmholtz Centre for Infection Research (HZI) 28336, Braunschweig, Germany; 2 Institute of Molecular Bacteriology, TWINCORE Centre for Experimental and Clinical Infection Research, a joint venture of the Hannover Medical School (MHH) and the HZI 274069, Hannover, Germany; 3 Cluster of Excellence RESIST (EXC 2155), Hannover Medical School 9177, Hannover, Germany; 4 Department of Clinical Microbiology, Copenhagen University Hospital—Rigshospitalet 683301, Copenhagen, Denmark; 5 Institute of Preventive Veterinary Medicine, Sichuan Agricultural University 506176, Chengdu, Sichuan, China; 6 Research Center of Avian Diseases, College of Veterinary Medicine, Sichuan Agricultural University 506176, Chengdu, Sichuan, China; 7 Key Laboratory of Animal Disease and Human Health of Sichuan Province, Chengdu, Sichuan, China; 8 Central Facility for Microscopy, Helmholtz Centre for Infection Research 28336, Braunschweig, Germany; Center for Microbial Dynamics & Infection, Atlanta, Georgia, USA

**Keywords:** motility, O-antigen, *P. aeruginosa*, pathogenicity

## Abstract

*Pseudomonas aeruginosa* is a versatile, opportunistic pathogen, well-known for its adaptability to the challenging conditions encountered during infection of the human host. In chronic biofilm-associated infections, variations in motility and LPS structure are commonly observed adaptive traits. In this study, we identified differences in the expression of the O-specific antigen (OSA) biosynthetic cluster as a key driver of motility variation among a large number of clinical PA14 clade isolates. Despite retaining flagellar presence and function, OSA-deficient mutants exhibited increased hydrophobicity, which enhanced surface attachment and, in turn, reduced surface motility while promoting biofilm formation. The modified lipopolysaccharide structure with a lack of OSA also resulted in increased serum sensitivity, reduced phagocytic uptake, increased aminoglycoside resistance, and enhanced expression of the type III secretion system. These findings suggest that the commonly observed loss of OSA in *P. aeruginosa* isolates recovered from chronically infected cystic fibrosis lungs may be selectively advantageous. Importantly, we demonstrate that clinically relevant phenotypes influenced by the presence of OSA, such as motility and antibiotic resistance, become detectable only under test conditions that promote bacterial attachment. Similarly, the role of OSA expression in bacterial pathogenicity is conditional, with neither its presence nor its absence uniformly favoring infection. Together, our findings support a model in which OSA expression may be beneficial during early infection, whereas reduced OSA expression may promote traits associated with persistence at later stages.

**IMPORTANCE:**

Loss of the O-specific antigens (OSA) in *Pseudomonas aeruginosa* isolates recovered from chronically infected cystic fibrosis lungs is a commonly observed phenotype that may confer a selective advantage to this opportunistic pathogen. In this study, we demonstrate that *P. aeruginosa* strains lacking OSA become more adhesive, limiting their motility but enhancing biofilm formation. Furthermore, OSA-deficient strains exhibit increased serum sensitivity, while they are phagocytosed less, express elevated type III secretion system genes, and show enhanced antibiotic resistance. Our findings suggest that altered OSA expression may contribute to stage-dependent infection phenotypes, where reduced OSA expression during later stages drives biofilm formation and antibiotic resistance, thereby enabling persistence.

## INTRODUCTION

*Pseudomonas aeruginosa* is an opportunistic human pathogen of concern due to its high prevalence in hospital-acquired infections, frequent multidrug resistance, and associated high mortality. Infections may be acute or progress into biofilm-associated, chronic infections, which are especially challenging to treat (1, 2). To address the critical medical needs and to provide the basis for the development of new therapeutic strategies that enhance bacterial killing or attenuate virulence, a deeper understanding of the pathogenicity of *P. aeruginosa* under physiological conditions and of the distinct molecular mechanisms that drive antimicrobial resistance, tolerance, and persistence is essential (3).

For many bacteria, motility is a key pathogenicity feature as it enables pathogens to move into favorable environments, attach to surfaces, and initiate infection. Motility, such as flagella-driven swimming or pilus-mediated twitching, facilitates initial attachment to host tissue. Beyond initial attachment, motility helps bacteria to evade immune responses or spread to new tissues. It also plays a key role in the formation of biofilms, which allow bacteria to form dense, protected communities on surfaces where they are less susceptible to antibiotics and the host immune system (4–7). The clinical outcome of an infection ultimately depends on the interaction between the bacterium and its host. Host cells detect pathogen-associated molecular patterns (PAMPs) through pattern-recognition receptors (PRRs), triggering cytokine secretion that activates immune defenses (8, 9). Expression of factors such as flagellin (major flagellar protein) or lipopolysaccharides (LPS), both of which are major PAMPs, therefore, not only benefit the bacteria but also allow the host to mount an immune response. We previously showed that the conversational communication at the host-pathogen interface is complex and dynamic. Beyond the presence of PAMPs triggering immune responses, motility itself has been linked to the induction of a specific *P. aeruginosa* gene expression program upon contact with host cells, which activates additional host immune modulators, promoting, e.g., phagocytic uptake (10).

In this study, we investigated how *P. aeruginosa* balances the tradeoff between motility and flagellin exposure as a PAMP. We stratified a previously well-characterized collection of clinical *P. aeruginosa* isolates according to their motility phenotypes and leveraged previously recorded transcriptional profiles (11). We identified variability in the expression of the O-specific antigen (OSA) biosynthetic gene cluster as a key player in modulating motility and propose a model in which the expression of the LPS O-antigen is crucial during the initial stages of the infection, as it promotes motility and protects the bacteria from the innate immune response. However, as the infection progresses, downregulation of OSA expression benefits *P. aeruginosa* by enhancing attachment, biofilm density, and antimicrobial resistance. This conditional and stage-dependent model in which OSA expression is advantageous during acute but disadvantageous during chronic infection constitutes the unifying framework for the phenotypic characterization performed throughout our study.

## RESULTS

### Diverse motility phenotypes in clinical *P. aeruginosa* isolates are linked to the regulation of O-antigen biosynthesis genes

**Fig 1:**
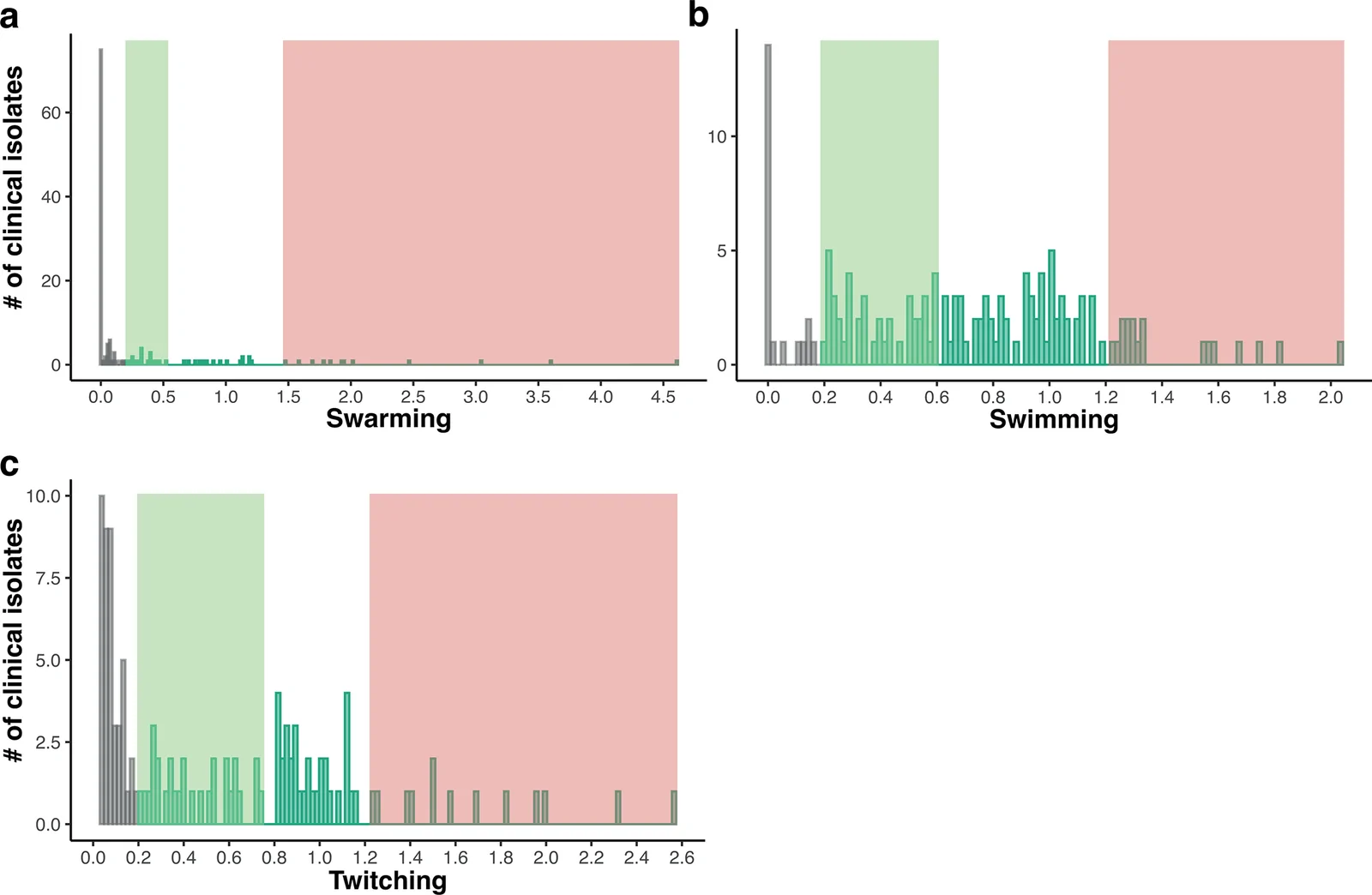
Swarming, swimming, and twitching motility of 144 clinical isolates. The relative distributions of clinical isolates exhibiting various swarming (**a**), swimming (**b**), and twitching (**c**) capabilities are depicted. The clinical isolates are sorted on the *x*-axis according to their swarming and twitching areas and swimming zone diameters normalized to PA14 WT. Isolates with low motility are highlighted in green and those with high swarming, swimming, or twitching motility in red. Non-motile isolates are shown in gray. The thresholds delineating the colored zones were set to reflect the asymmetric distribution of normalized values around 1, with the spread of values above 1 being considerably larger than below, and serve as a visual guide to distinguish isolates with notably altered motility phenotypes. For each motility phenotype, the low- and high-motility groups were defined independently.

To unravel new candidate genes involved in the modulation of *P. aeruginosa* motility, we selected 144 *P. aeruginosa* clinical isolates belonging to the PA14 clade and tested their swimming, swarming, and twitching motility using standardized assays (11). Transcriptional profiles of all isolates grown in LB to an OD_600_ of 2 were available from previous work (11). As shown in Fig. 1, the clinical isolates were categorized into non-motile, low-motile, and high-motile groups. No relevant correlations were found between the different motility types (Fig. S1). Two-thirds (96/144) of the clinical isolates exhibited no swarming motility under our screening conditions (Fig. 1a). We, therefore, focused our subsequent analysis on swimming and twitching motility, both of which displayed broad variability among the clinical isolates (Fig. 1b and c).

**Fig 2:**
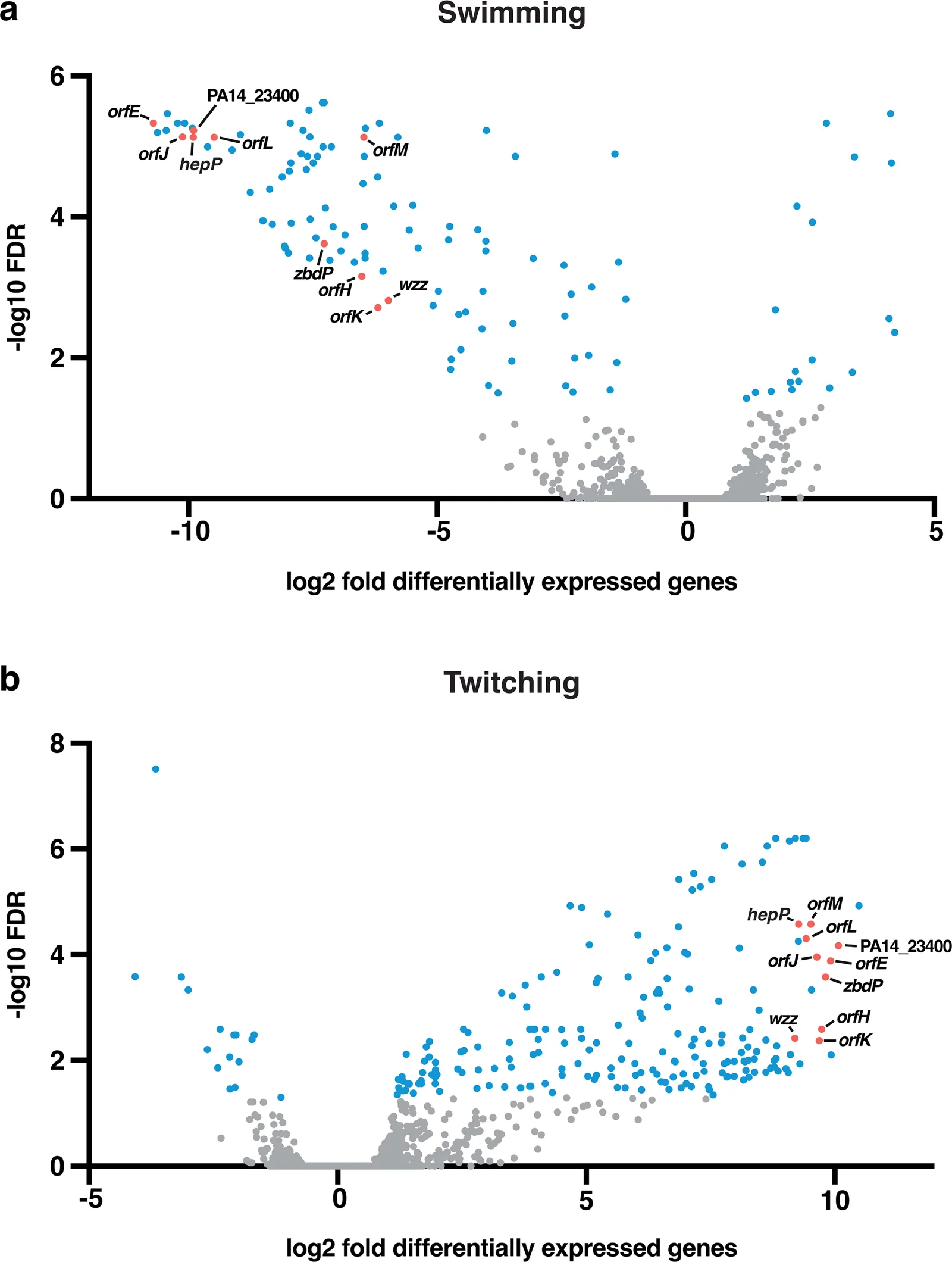
Differentially expressed genes in groups with high vs low motility phenotypes. Volcano plots showing differential gene expression in clinical isolates with low swimming (**a**) or low twitching (**b**) motility compared to isolates with high swimming or high twitching motility, respectively. Genes were considered differentially regulated if they displayed a log2 fold change of ≥|1.5| (*x*-axis) with a false discovery rate (FDR) of <0.05 (*y*-axis). Differentially regulated genes are highlighted in blue; genes involved in O-specific antigen biosynthesis are highlighted in red.

We next compared the transcriptomes of isolates with low motility (highlighted in green) to those with high motility (highlighted in red). Non-motile isolates were excluded from our analysis, as their lack of motility most likely results from the loss of genes involved in the production of structural components required for the different types of motility. We found 119 differentially expressed genes (DEGs) between low- and high-swimming isolates (Fig. 2a; Table S1) and 212 DEGs between low- and high-twitching isolates (Fig. 2b; Table S2). For each motility phenotype, isolates were ranked and assigned to low- and high-motility groups independently. Accordingly, the strain subsets used for the swimming and twitching transcriptome comparisons were defined separately. Among the DEGs shared between both data sets was a gene cluster involved in LPS O-antigen biosynthesis. This cluster (PA14_23360–PA14_23470) comprises 12 consecutive genes that were downregulated in isolates with low swimming motility (Fig. 2a; Table S3) but strongly upregulated in isolates with low twitching motility (Fig. 2b).

The LPS of *P. aeruginosa* is an integral component of the outer membrane and consists of three domains: the lipid A, the core oligosaccharide, and the O-specific antigen (OSA). Most strains produce two types of O-polysaccharides: the homopolymeric common polysaccharide antigen (CPA; formerly A band) and the heteropolymer OSA (formerly B band) (12, 13). PA14 does not produce CPA but synthesizes both very long and short OSA chains (14). Comparative genomic analysis between the 2 *P. aeruginosa* type strains PA14 and PAO1 revealed that 5 O-antigen biosynthetic genes are shared, while 7 genes are unique to PA14 and 12 to PAO1 (Fig. S2).

### Functional roles of the O-antigen biosynthesis genes in *P. aeruginosa*

**Fig 3:**
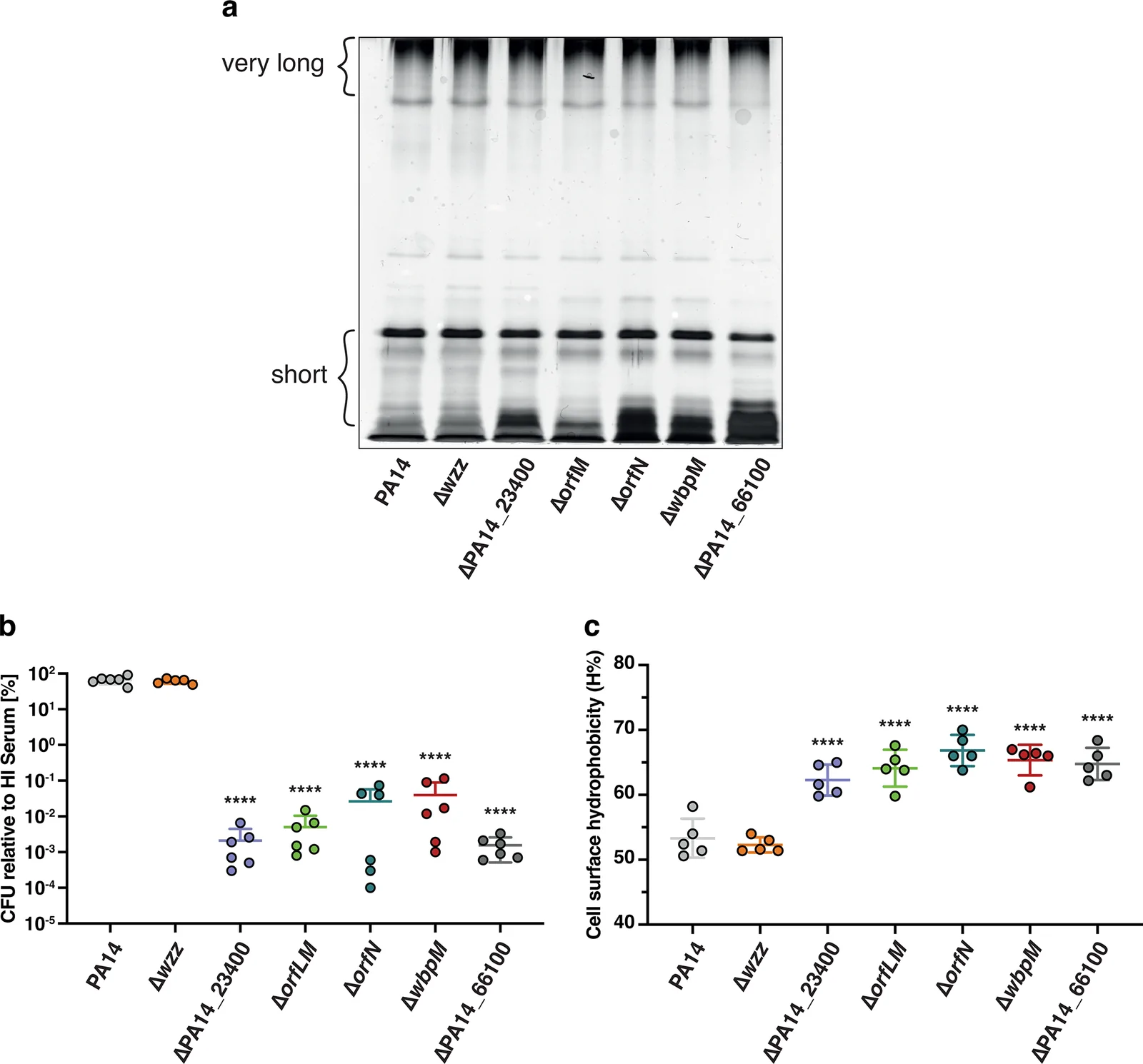
Functional characterization of O-specific antigen biosynthesis mutants. (**a**) Silver-stained 12.5% SDS-PAGE used to characterize short and very long chains of the O-specific antigen in PA14 and the indicated deletion mutants. ΔPA14_66100, which lacks the O-antigen ligase required to connect the O-antigen to the lipid A-core, is shown as a control. (**b**) Complement sensitivity of PA14 and the indicated deletion mutants, determined as CFUs after 30 min incubation in human blood serum and normalized to CFUs obtained in heat- inactivated (HI) serum. (**c**) Cell surface hydrophobicity (H%) was determined by the MATH test. The knock-out mutants exhibited an increase in cell surface hydrophobicity of at least 8%. Mean ± SD of five biological replicates are shown. ****P < 0.0001, one-way analysis of variance (ANOVA) (*post hoc* test: Dunnett).

To investigate the functional roles of individual genes within the OSA biosynthetic cluster, we generated deletion mutants of *wzz*, PA14_23400, *orfM, orfN,* and *wbpM* using a Cas9-assisted genetic engineering method (15), as well as chromosomally complemented strains for each mutant. We first analyzed the LPS phenotypes of the deletion and complemented mutants by silver-stained SDS-PAGE (Fig. 3a). As a control, we used the O-antigen ligase mutant ΔPA14_66100 (*waaL*) which lacks the ligase responsible for the final step of covalently attaching the mature O-antigen polysaccharide to the lipid A-core, without impairing O-antigen biosynthesis. This phenotype is, therefore, distinct from that of a *wbpL* mutant, in which the O-specific antigen LPS biosynthesis is abolished (16). The overall pattern of very long OSA chains appeared similar in all mutants. However, within the short-chain region, a distinct cluster of bands present in PA14, Δ*wzz*, and ΔPA14_23400 was markedly reduced in the Δ*orfM,* Δ*orfN,* and Δ*wbpM* mutants and almost absent in ΔPA14_66100. Deletion of PA14_23400 resulted in only minor alterations, while no changes were observed in the deletion of the O-antigen chain-length regulator encoding gene *wzz* (17, 18). These results indicate that OSA biosynthesis mutants exhibit an altered distribution and reduced abundance of short OSA chains rather than a complete loss of O-antigen. Importantly, chromosomal complementation of each deletion mutant restored the wild-type OSA pattern as confirmed by silver-stained SDS-PAGE, demonstrating that the observed LPS alterations are directly attributable to the respective gene deletions (Fig. S3).

Given that OSA plays an important role in resistance against the human complement system, we examined the serum resistance of the deletion mutants in comparison to PA14 WT. Heat-inactivated serum served as a negative control. Both the PA14 WT and the Δ*wzz* mutant exhibited high survival rates exceeding (~60%–70%). In contrast, all other mutants showed survival levels below 0.01% upon complement exposure (Fig. 3b).

To further evaluate whether the absence of OSA leads to altered cell surface hydrophobicity, we tested the deletion mutants using the microbial adhesion to hydrocarbons (MATH) assay (19, 20). With the exception of the Δ*wzz* mutant, which retained WT-like OSA production, all other mutants demonstrated a significant increase in cell surface hydrophobicity, with values ranging from 62% to 67%, compared to a mean value of 54% for the PA14 WT (Fig. 3c).

### Lack of OSA impairs motility on solid surfaces but not swimming velocity in liquid media

**Fig 4:**
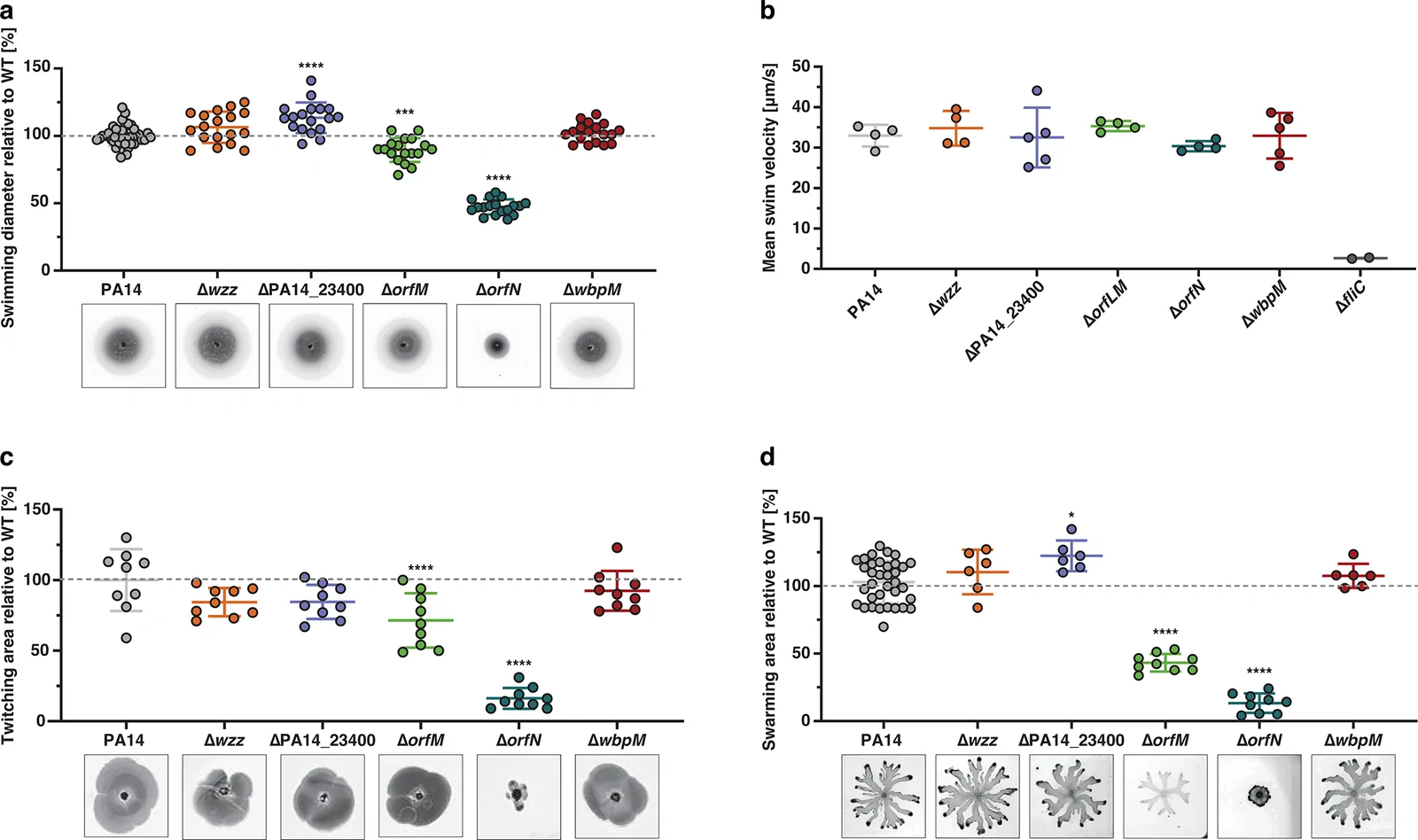
Influence of the presence of O-specific antigens on motility phenotypes. (**a**) Swimming diameter of the indicated deletion mutants relative to the WT (in %) after 18 h of incubation on 0.3% swimming agar plates at 37°C. Representative swimming agar plates are shown under the graph. (**b**) Mean swimming velocity of individual cells in liquid medium. For each strain, more than 165 cells with a minimum velocity of 15 μm/s were tracked across at least four independent videos. (**c**) Twitching motility of the indicated deletion mutants relative to the WT (in %) after 48 h of incubation on 1.5% twitching agar plates at 37°C. Representative twitching plates are shown under the graph. (**d**) Swarming motility of the indicated deletion mutants relative to the WT (in %) after 16 h of incubation on 0.5% swarming agar plates at 30°C under humid atmosphere. Representative swarming areas are shown under the graph. *P < 0.05, ***P < 0.001, ****P < 0.0001, one-way analysis of variance (ANOVA) (*post hoc* test: Dunnett). See Fig. S4 for complementation of swimming, swarming, and twitching motilities.

Having established the LPS phenotypes of the OSA biosynthesis mutants, we next investigated their role in PA14 motility. Deletion of *wzz* did not alter swimming motility compared to WT. This gene shares 56% amino acid identity with its functional homolog *wzz1* in PAO1 (PA3160), which regulates the production of long OSA chains that are absent in PA14 (14, 17, 18). Likewise, deletion of *wbpM*, encoding a nucleotide sugar epimerase/dehydratase, showed no difference from WT. In contrast, deletion of *orfM*, encoding a putative NAD-dependent epimerase/dehydratase, and *orfN*, encoding a putative group 4 glycosyl-transferase, resulted in significantly reduced swimming motility, with the Δ*orfN* mutant showing the strongest defect (Fig. 4a). Complementation of *orfM* restored and complementation with *orfN* partially restored the WT phenotype (Fig. S4a). Notably, deletion of PA14_23400, encoding a hypothetical protein, led to a slight but significant increase in swimming motility relative to the WT, which was abolished in the complemented mutant (Fig. 4a; Fig. S4a). To ensure that these differences were not caused by growth defects, we compared the growth rates of all deletion mutants to that of their parental strain in LB. No significant differences were observed (Fig. S5).

To assess whether the flagellum was present and functional in the deletion mutants, we performed negative staining transmission electron microscopy (TEM) (Fig. S6a) as well as scanning electron microscopy (SEM) (Fig. S6b) to visualize the flagella. Intact and extended flagella were observed for all OSA biosynthesis mutants, indicating that flagellar production and assembly are functional. We then tracked individual cells and measured their swimming speed at the single-cell level in liquid media (19). No significant differences were found between any of the deletion mutants and the parental strain, demonstrating that the flagella are functional and energized (Fig. 4b).

Our transcriptome analysis identified the gene cluster PA14_23360–PA14_23470 as differentially expressed not only between groups of clinical isolates with high vs low swimming motility but also between groups with high vs low twitching motility (Fig. 2b). We, therefore, also examined twitching and swarming motility in the OSA biosynthesis mutants. In the twitching assay, deletion of *wzz*, ΔPA14_23400, and *wbpM* did not result in statistically significant differences compared to the PA14 WT although Δ*wzz* and ΔPA14_23400 showed a slight tendency toward reduced twitching, which was restored to WT levels in the complemented Δ*wzz* and Δ*wbpM* mutant and even exceeded the levels of the WT in the complemented ΔPA14_23400 mutant (Fig. 4c; Fig. S4b). In contrast, deletion of *orfM* and Δ*orfN* significantly decreased the twitching area, with the Δ*orfN* mutant exhibiting the strongest defect and showing only minimal residual twitching (Fig. 4c). The complemented Δ*orfM* mutant fully and the complemented Δ*orfN* partially restored WT twitching levels (Fig. S4b).

A similar pattern was observed for swarming motility. Deletion of *wzz* and *wbpM* did not affect swarming compared to the WT, whereas the ΔPA14_23400 mutant displayed a slight but significant increase in swarming motility, in line with its mildly enhanced swimming phenotype, which was not restored to WT levels in the complemented mutant (Fig. S4c). Deletion of *orfM* and *orfN* again resulted in markedly reduced swarming, with Δ*orfN* showing the most pronounced decrease in surface spreading (Fig. 4d). The WT phenotype was fully restored in the complemented Δ*orfM* and partially in the complemented Δ*orfN* mutant (Fig. S4c).

Taken together, these results demonstrate that several genes within the O-antigen biosynthesis cluster contribute to surface-associated motility in PA14. In particular, Δ*orfM* and especially Δ*orfN* are required for normal levels of swimming, twitching, and swarming motility, whereas Δ*wzz* and Δ*wbpM* do not measurably affect these phenotypes under the conditions tested. ΔPA14_23400, by contrast, shows a slight but consistent increase in motility.

### Lack of OSA influences biofilm maturation and architecture

**Fig 5:**
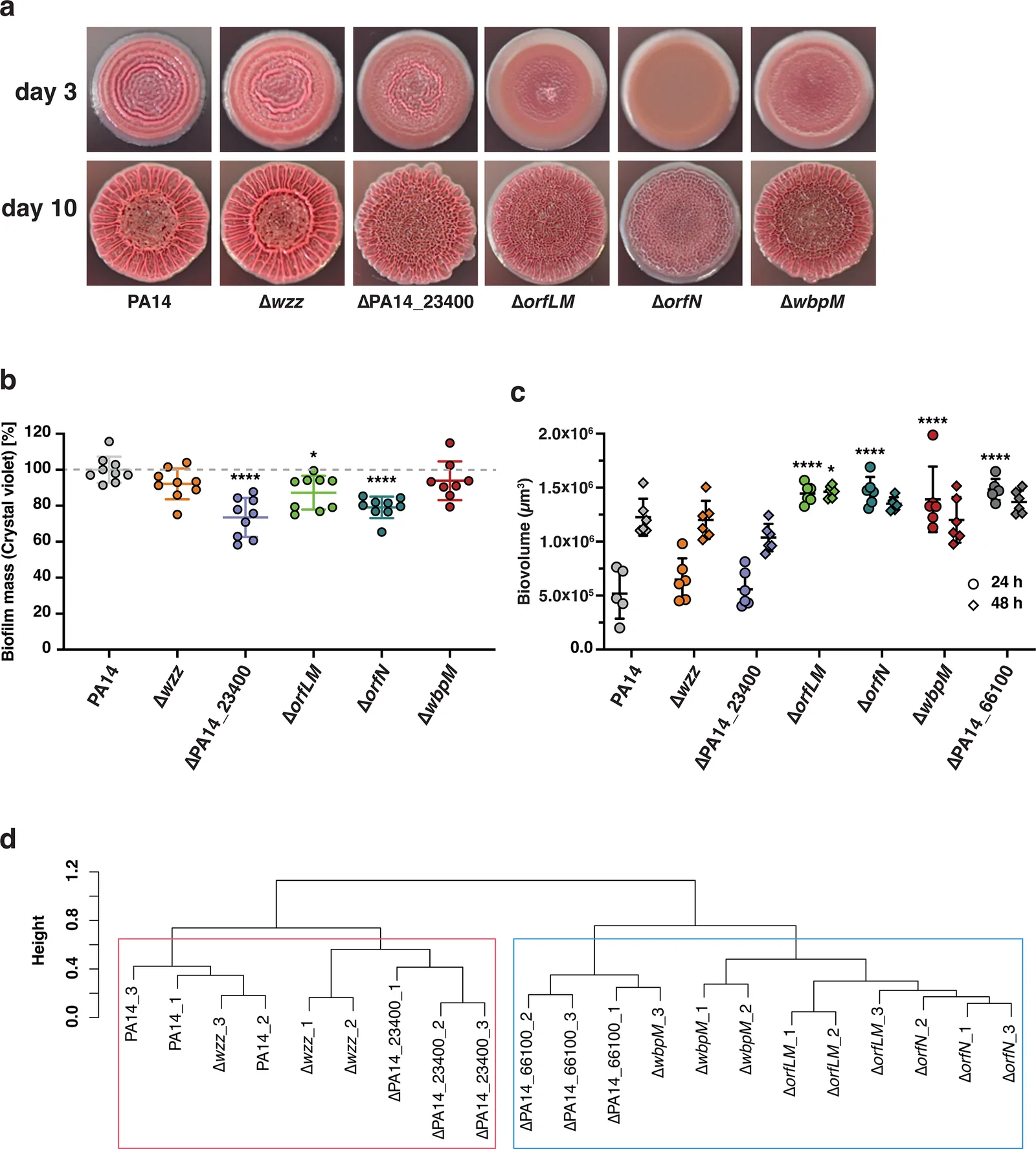
Biofilm formation in the O-specific antigen mutants. (**a**) Colony biofilms of PA14 WT and indicated O-specific antigen biosynthesis mutants grown on agar medium containing Congo red and Coomassie blue for 3 and 10 days are depicted. (**b**) Quantification of the biomass relative to that of the WT (in %) in the indicated O-specific antigen biosynthesis mutants using the crystal violet assay of biofilms grown in 96-well plates for 24 h. *P < 0.05, ****P < 0.0001, one-way ANOVA (*post hoc* test: Dunnett). (**c**) PA14 and indicated deletion mutant biofilms were grown for 24 and 48 h in 96-well plates, and biovolumes were determined following image acquisition with confocal laser scanning microscopy. Asterisks indicate statistically significant differences in biovolume between the mutant and PA14 WT under the same condition (*P < 0.05, ****P < 0.0001, one-way ANOVA [*post hoc* test: Dunnett]). (**d**) Cluster analysis of three biological replicates of PA14 (PA14_1, PA14_2, and PA14_3) and its respective O-specific antigen deletion mutant biofilms after 48 h of incubation using seven parameters: biovolume (µm^3^), area-to-volume ratio (µm^−1^), horizontal and vertical spreading (µm^2^), mean thickness (µm), biofilm roughness, and substrate coverage at the bottom of the well. The clusters were defined by hierarchical clustering with a cutoff of 0.5.

Given the observed deficiencies in swimming, twitching, and swarming motility among the O-antigen biosynthesis mutants, we next examined whether alterations in OSA structure affect colony biofilm development. When grown on Congo red-containing agar, both the PA14 WT and the Δ*wzz* mutant displayed intense red staining and pronounced wrinkle formation, indicative of robust extracellular matrix production (Fig. 5a). The ΔPA14_23400 and Δ*wbpM* mutants were similar in appearance although staining and wrinkle formation were reduced. In contrast, the Δ*orfLM* showed paler pink colonies, and wrinkle formation was reduced. The Δ*orfN* mutant exhibited the strongest phenotype, remaining pink even after extended incubation. In addition to these morphological differences, all OSA deletion mutants formed smaller microcolonies overall than the WT (Fig. S7a).

We then assessed early-stage biofilm formation using the crystal violet assay. Although all mutants produced slightly less biofilms than the PA14 WT, statistically significant reductions were observed only for Δ*orfN*, ΔPA14_23400, and Δ*orfLM* mutants (Fig. 5b). Of note, the ΔPA14_23400 mutant generated the lowest biofilm mass (73.5% of WT) despite displaying only minor defects in twitching motility.

To further examine biofilm development, we used confocal laser scanning microscopy (CLSM) to analyze the biovolume and three-dimensional architecture of biofilms after 24 and 48 h of incubation (Fig. 5c; Fig. S7b). As a control, we included the O-antigen ligase-deficient mutant, ΔPA14_66100, which produces the sugar components of OSA but cannot attach them to the lipid A-core molecule. To compare the overall biofilm architecture of the different strains at the mature biofilm stage, we performed a cluster analysis based on seven quantitative parameters extracted from the CLSM data sets after 48 h of growth (Fig. 5d). Overall, two main clusters were identified using a hierarchical clustering cutoff of 0.5. At 48 h, Δo*rfLM*, Δ*orfN*, Δ*wbpM*, and ΔPA14_66100 clustered separately from PA14 WT, Δ*wzz,* and ΔPA14_23400, indicating distinct mature biofilm architectures. Notably, the strains in this cluster had already reached high biovolumes after 24 h, whereas PA14 WT, Δ*wzz*, and ΔPA14_23400 reached similar biovolumes only after 48 h (Fig. 5c). In contrast, PA14 WT, Δ*wzz,* and ΔPA14_23400 strains reached similar high biofilm volumes only after 48 h of growth. At this later time point, the biofilm volumes of the other mutants (in particular Δ*orfN* and Δ*wbpM*) already tended to decrease. These findings suggest that the mutants lacking OSA are characterized by a faster initial biofilm development and earlier structural consolidation, whereas strains that express OSA display a slower and more progressive biofilm maturation pattern.

### The role of OSA on antibiotic resistance is conditional

In a previous machine-learning approach predicting antimicrobial resistance to four different antibiotics, genes from the O-antigen biosynthesis gene cluster were identified as potential contributors to *P. aeruginosa* resistance against ceftazidime, tobramycin, and meropenem (21). Furthermore, polycationic aminoglycosides are known to bind to the negatively charged components of the bacterial outer membrane, including phospholipids and OSA. This interaction displaces magnesium cations, destabilizes the outer membrane, and ultimately leads to enhanced permeability and aminoglycoside uptake (22, 23).

To assess the role of OSA in antimicrobial susceptibility, we compared the minimal inhibitory concentration (MIC) of the OSA biosynthesis mutants. The results obtained with Epsilometer tests (Etest) are shown in Table 1. The Δ*orfLM*, Δ*orfN,* and Δ*wbpM* mutants exhibited increased MIC values for amikacin, tobramycin, and ceftazidime, ranging from two- to fourfold compared to the PA14 WT. Δ*wbpM* additionally showed a ~2-fold increased MIC for levofloxacin. In contrast, the ΔPA14_23400 mutant displayed only minor MIC increase (about two- to threefold) across the antibiotics tested. However, MIC values determined by broth microdilution did not differ from those of the PA14 WT for any mutant or antibiotic (Table 1). These results indicate that the observed resistance phenotype of the O-antigen biosynthesis mutants becomes apparent only during growth on solid surfaces.

**TABLE 1:** Antibiotic susceptibility profiles of PA14 and O-specific antigen biosynthesis mutants^*a*^

Strain	Amikacin MIC (µg/mL)		Tobramycin MIC (µg/mL)		Ceftazidime MIC (µg/mL)		Levofloxacin MIC (µg/mL)	
	Etest	Broth microdilution assay	Etest	Broth microdilution assay	Etest	Broth microdilution assay	Etest	Broth microdilution assay
PA14 WT	2	1	0.75	1	0.5	1	0.38	0.125
Δ*wzz*	3	1	0.75	1	0.5	1	0.38	0.25
ΔPA14_23400	6	2	1.50	1	1	1	0.5	0.25
Δ*orfLM*	8	2	2	1	1.5	1	0.5	0.25
Δ*orfN*	8	2	2	1	1.5	1	0.5	0.25
Δ*wbpM*	12	1	2	1	1.5	1	0.75	0.125

^*a*^ MICs were determined using the Etest (on solid media) and the microbroth dilution assay (liquid media) for four different antibiotics.

**Fig 6:**
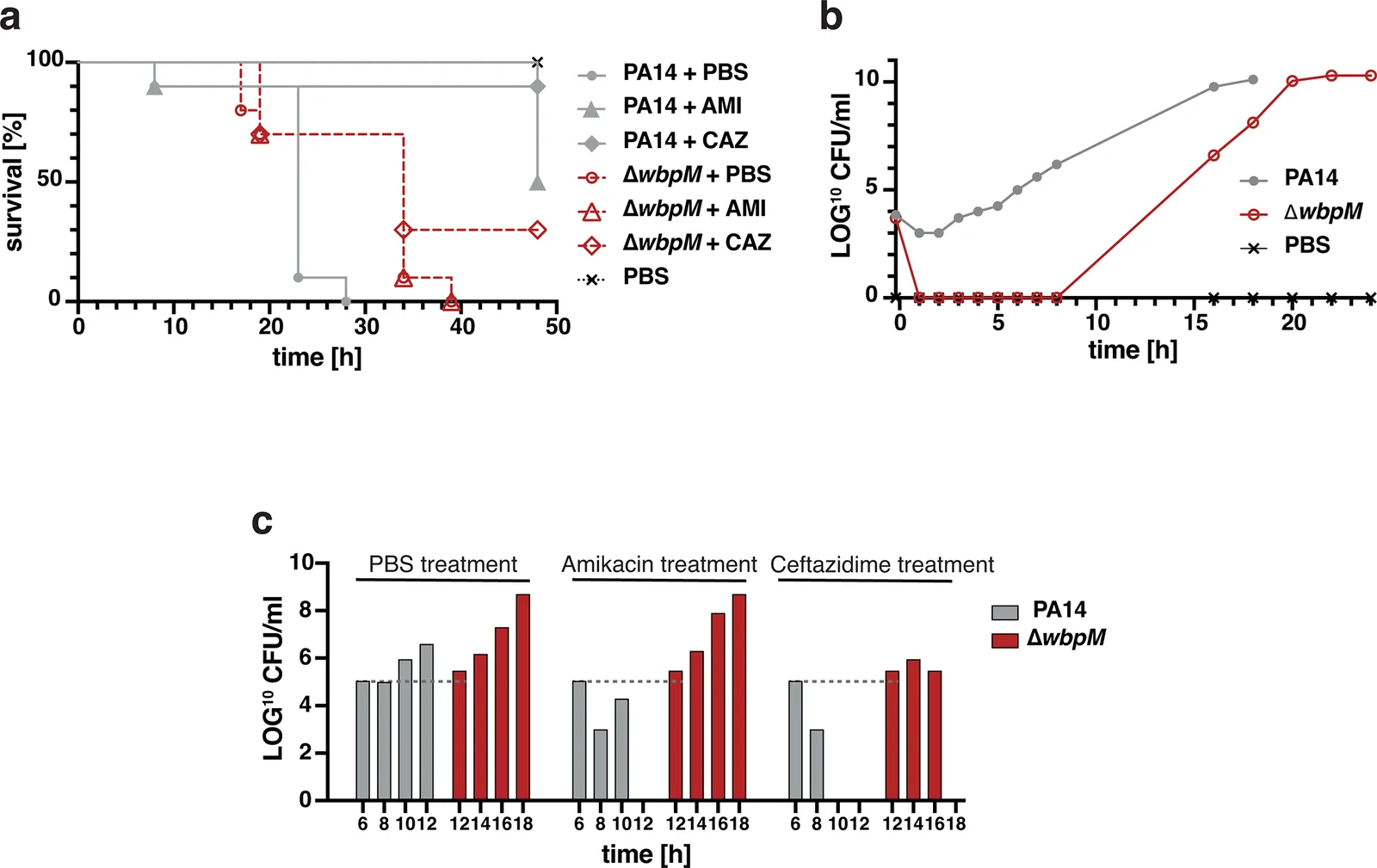
Evaluation of virulence and survival of O-specific antigen mutants in the *Galleria mellonella* infection model. (**a**) Survival (in %) of *G. mellonella* larvae infected with PA14 WT or the Δ*wbpM* mutant over 48 h. Data are representative of three independent biological replicates (*n* = 20 larvae per condition). For antibiotic treatment, PA14 WT-infected larvae were treated with PBS, amikacin (AMI), or ceftazidime (CAZ) 6 h post infection, while Δ*wbpM*-infected larvae were treated 12 h post-infection to account for differences in bacterial outgrowth. (**b**) Bacterial loads (CFUs per larva) of PA14 WT and Δ*wbpM* in hemolymph over 24 h. Data represent CFUs measured using pooled hemolymph from six larvae. (**c**) Bacterial loads in larvae infected with PA14 WT or Δ*wbpM* and treated with AMI or CAZ (10× MIC). PA14 WT-infected larvae were treated at 6 h post-infection and Δ*wbpM*-infected larvae at 12 h post-infection. Data represent CFUs measured using pooled hemolymph from six larvae. The gray dashed line indicates the bacterial load at the start of treatment.

We next investigated whether OSA contributes to antibiotic resistance during infection using the *Galleria mellonella* model (24). Given that WbpM is highly conserved, crucial for OSA biosynthesis, and broadly associated with antibiotic susceptibility on solid media (25, 26), we compared infections with PA14 WT and Δ*wbpM*. Infections with WT resulted in complete larval death within 28 h (Fig. 6a), whereas Δ*wbpM* caused delayed killing, with full mortality occurring by 39 h post-infection (Fig. 6a). With the exception of Δ*wzz,* the other OSA biosynthetic mutants ΔPA14_23400*,* Δ*orfLM,* and Δ*orfN* also clearly showed attenuated virulence in this model (Fig. S8).

To monitor bacterial proliferation during infection, we quantified CFU in the larval hemolymph. WT cells proliferated after a brief 2-h lag phase, reaching lethal levels by 18 h. In contrast, Δ*wbpM* was reduced below the detection limit for up to 8 h post-infection before resuming growth at a rate similar to that of WT, eventually reaching comparable bacterial loads by 22 h (Fig. 6b).

To evaluate the effect of OSA on treatment efficacy, infections were initiated with PA14 WT or Δ*wbpM* and then treated with amikacin or ceftazidime after allowing each strain to establish the infection (6 h for WT and 12 h for Δ*wbpM* to account for differences in early bacterial load). In WT infections, treatment with either antibiotic prolonged larval survival beyond 46 h post-infection (Fig. 6a) and clearance of the bacterial load within 6 h (amikacin) or 4 h (ceftazidime) (Fig. 6c). Ceftazidime was also effective against Δ*wbpM* infections although with lower efficiency, leading to 30% larval survival at 48 h and complete bacterial clearance after 18 h (Fig. 6a and c). However, treatment with amikacin had no effect on larval viability (Fig. 6a and c) and failed to clear Δ*wbpM* from the larvae (Fig. 6c). Since bacterial loads were reduced or cleared after treatment, lethality occurring around 48 h may reflect infection-associated host damage and/or excessive activation of innate immune responses rather than uncontrolled bacterial outgrowth (27–29). Altogether, these findings demonstrate that amikacin resistance of Δ*wbpM* is observed not only *in vitro* (Etest) but also *in vivo*.

### Lack of OSA reduces phagocytic uptake and enhances cytotoxicity

**Fig 7:**
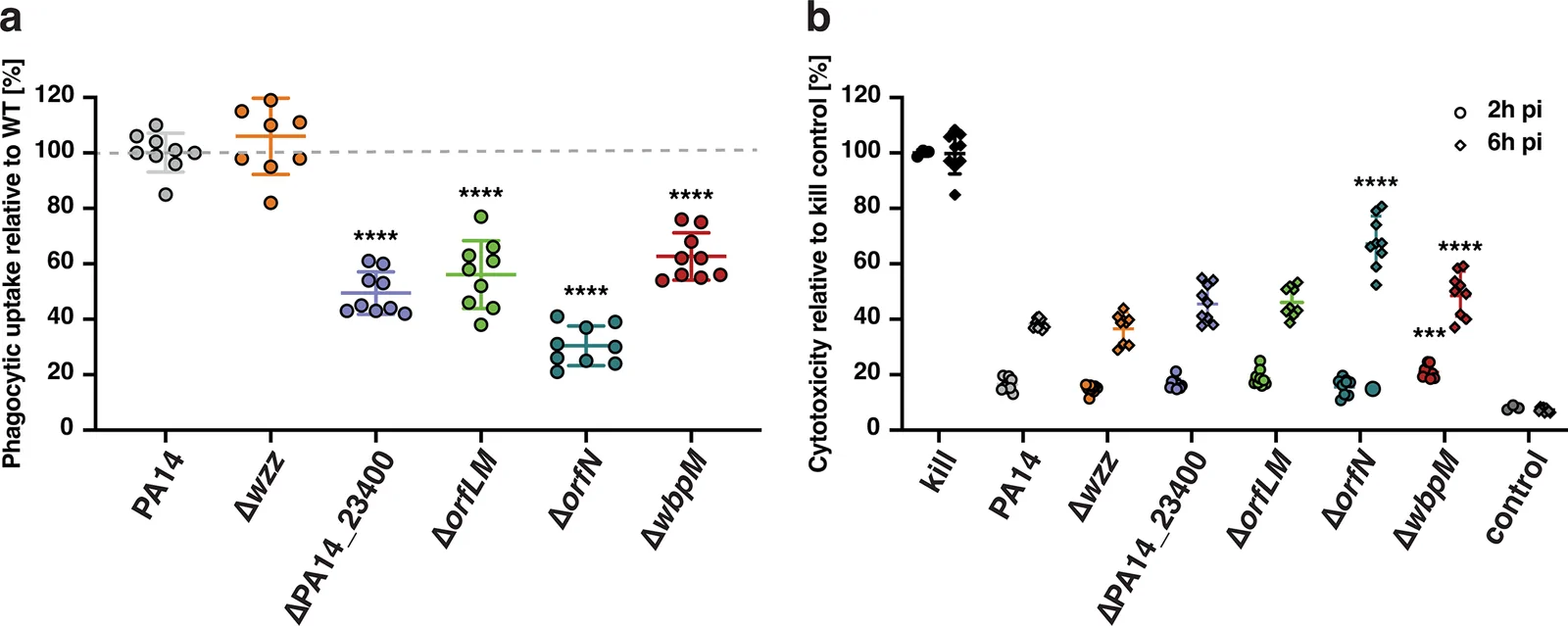
Cytotoxicity of the O-specific antigen mutants. (**a**) Phagocytic uptake of PA14 WT and the indicated deletion mutants by RAW264.7 macrophages 2 h post-infection at a multiplicity of infection (MOI) of 1. Intracellular CFUs were normalized to PA14 WT. (**b**) LDH as a measure of cytotoxicity 6 h post-infection at an MOI of 1. Untreated macrophages served as negative control and macrophages lysed with lysis buffer as killing control, to which values were normalized. **P < 0.01, ***P < 0.001, ****P < 0.0001; significant differences between the deletion mutants and PA14 WT were determined by a one-way ANOVA (*post hoc* test: Dunnett).

O-antigens have previously been described as protective structures against phagocytosis in a variety of organisms (30–32). To assess the impact of OSA deficiency on the interaction of *P. aeruginosa* with macrophages, we performed gentamicin protection assay using RAW264.7 cells (Fig. 7a) (33). With the exception of the Δ*wzz* mutant, which exhibited intracellular CFU counts comparable to those of the PA14 WT, all other OSA biosynthesis mutants showed significantly reduced intracellular CFUs at 2 h post-infection, reaching only 30%–63% of WT levels. We also measured cytotoxicity by determining LDH release 6 h post-infection (Fig. 7b). The LDH levels in the supernatants of macrophages infected with the Δ*wzz,* ΔPA14_23400, or Δ*orfLM* mutants did not differ significantly from those infected with WT. In contrast, the Δ*orfN* and Δ*wbpM* mutants induced significantly higher LDH levels despite their markedly lower intracellular bacterial counts.

These results indicate that while OSA contributes to resistance against phagocytic uptake, its absence may lead to increased cytotoxic effects on macrophages, suggesting distinct roles of OSA in host-pathogen interactions.

### Expression of the O-antigen biosynthesis gene cluster is downregulated during *in vivo G. mellonella* infections

While the presence of the OSA is important for establishing full virulence and motility, the loss of these antigens appears to promote phenotypes that may become relevant in later stages of infection, such as biofilm formation and antibiotic resistance. Of note, the O-antigen biosynthetic gene cluster exhibits elevated expression levels during exponential phase of growth while expression levels decrease when entering late-exponential and stationary growth phases (34) (Fig. S9). To investigate how OSA expression changes during infection, we infected *G. mellonella* larvae and recorded an *ex vivo* transcriptional profile of PA14 WT 20 h post-infection. Differentially expressed genes were identified by comparing this *ex vivo* transcriptome to that of PA14 grown *in vitro* to an OD_600_ of 2.0 in LB medium.

**Fig 8:**
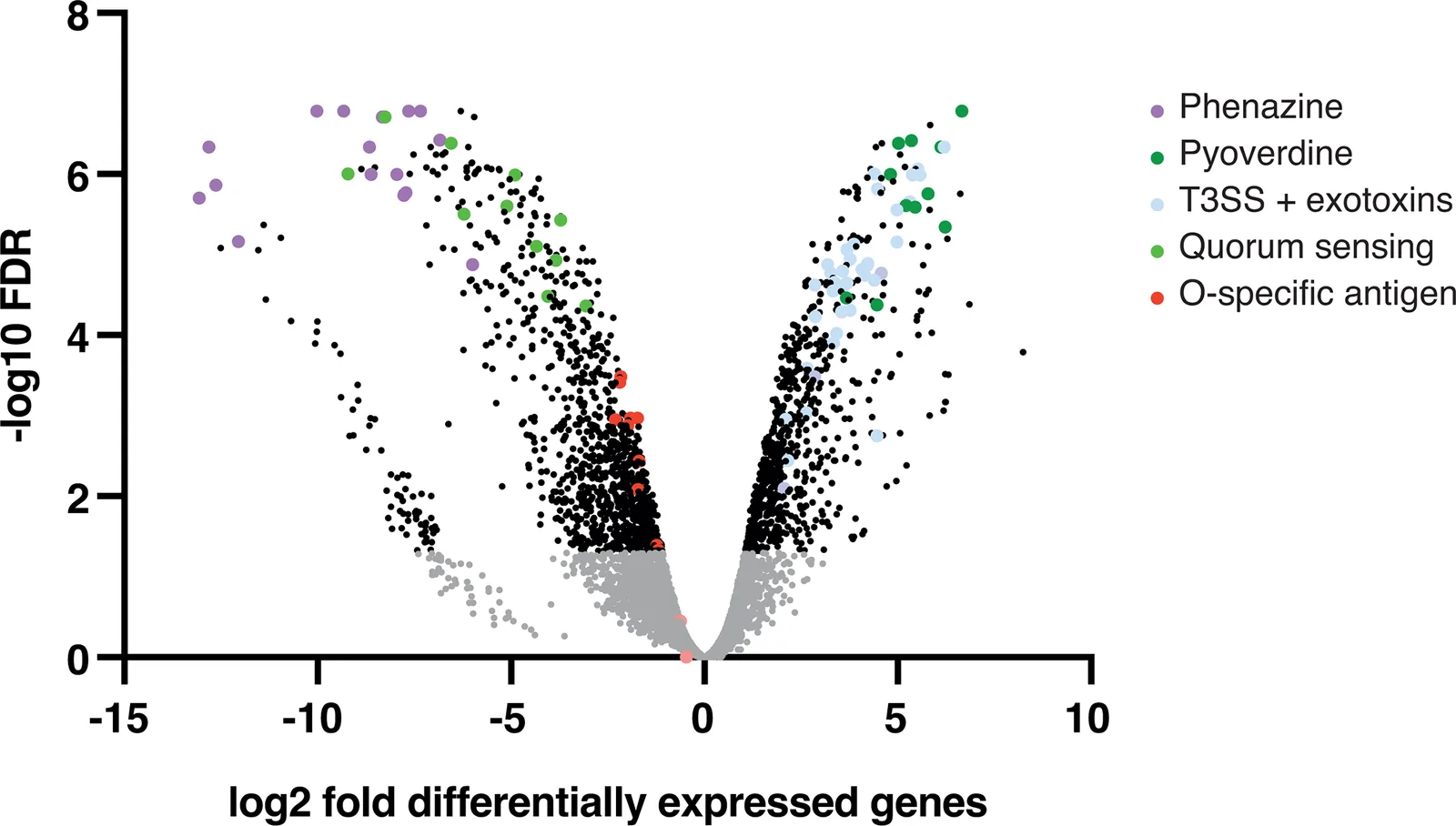
*Ex vivo* transcriptional profiling. *Ex vivo* transcriptome of PA14 recovered from *G. mellonella* larvae 20 h post-infection compared to PA14 grown in LB medium to an OD_600_ of 2. Differential expression was assessed using glmTreat (R package edgeR) with a log2 fold change threshold of |1.5|; genes with an FDR ≤ 0.05 were considered differentially regulated (*y*-axis). Differentially regulated genes are highlighted in black, and genes involved in phenazine biosynthesis, pyoverdine biosynthesis, the T3SS, quorum sensing, and O-specific antigen biosynthesis are highlighted in the indicated colors. RNA-seq was performed with *n* = 2 biological replicates per condition.

As expected, genes of the type three secretion system (T3SS) and genes involved in pyoverdine production were significantly upregulated at late stages of *G. mellonella* infection. Furthermore, 10 genes of the O-antigen biosynthetic gene cluster, including *orfKHEJLMN*, PA14_23400, *zbdP,* and *hepP*, were significantly downregulated *in vivo*, with log2 fold changes greater than 1.5 and FDR values <0.05 (Fig. 8). *wzz* and *wbpM* exhibited a trend toward downregulation as well although these changes did not reach statistical significance (Table S4).

These findings support the hypothesis that while OSA contributed to early infection processes, downregulation of the biosynthetic gene cluster may become advantageous as the infection progresses, potentially reflecting a transition toward stationary-phase or biofilm-like gene expression behavior.

## DISCUSSION

The opportunistic pathogen *P. aeruginosa* undergoes characteristic evolutionary adaptation during chronic respiratory tract infections (35). In patients with cystic fibrosis, chronic infections result in consistently observed phenotypic changes, including loss of motility and alterations in LPS structure. Loss of OSA is a well-described phenotype of CF isolates and results in a transition from smooth to rough LPS (12, 36, 37). However, the functional consequences of OSA loss and its role in infection dynamics remain incompletely understood.

Our data reveal that reduced OSA expression in clinical PA14 clade isolates diminishes swimming motility on semi-solid surfaces, even in the presence of a functional flagellum. Importantly, single-cell swimming velocity in liquid media remained unaffected, indicating that OSA does not influence flagellar function *per se* (38). Instead, the absence of OSA increases surface hydrophobicity (20), which promotes surface attachment and restricts motility on surfaces (39, 40). This increased hydrophobicity also accelerates biofilm maturity, as hydrophobic interactions stabilize bacteria-surface attachment and promote closer packing within biofilms (41). Previous studies have shown that increased cell surface hydrophobicity correlated with enhanced adherence to lung epithelial cells, suggesting a colonization advantage for hydrophobic strains *in vivo* (42, 43).

A central finding of the study is that the phenotypic consequences of OSA loss are conditional. PA14 strains lacking OSA exhibited enhanced resistance to aminoglycosides and ceftazidime, but this phenotype was only detectable on solid media (Etest) and in the *G. mellonella in vivo* model but not in conventional broth microdilution assays. This observation has important clinical implications, as it suggests that standard antimicrobial susceptibility testing may fail to detect resistance phenotypes that are relevant during surface-associated growth and biofilm infections. The conditionality of antibiotic resistance, together with increased biofilm and reduced phagocytic uptake, likely contributes to the selective advantage of rough LPS variants during chronic CF infections.

Beyond antibiotic resistance, OSA-deficient strains displayed additional phenotypes relevant to host-pathogen interaction. Phagocytic uptake by macrophages was reduced, while cytotoxicity was increased in several OSA mutants, likely through enhanced expression of the type III secretion system (T3SS). Previous studies have shown that O-antigen length can modulate the expression of the T3SS in *Salmonella enterica*, *Shigella flexneri,* as well as *P. aeruginosa* (44–46), and T3SS activity is well-established as a key determinant of *P. aeruginosa* virulence (47). Furthermore, loss of OSA may confer increased resistance to bacteriophages, as O-antigens serve as key entry points for many phages (48, 49).

Despite the advantages, OSA-deficient mutants were clearly attenuated in the *G. mellonella* model. This apparent paradox—enhanced persistence traits yet reduced acute virulence—is consistent with our previous finding of a positive correlation between expression of OSA biosynthesis genes (*orfN* and *wbpM*) and virulence in clinical *P. aeruginosa* isolates (24). The reduced virulence of OSA-deficient strains likely reflects the importance of OSA for early immune evasion during infection establishment, as has been previously demonstrated in *Salmonella* during apical intestinal epithelial invasion (50) and *G. mellonella* infections (51). Consistent with this interpretation, our *ex vivo* transcriptomic data show that the OSA biosynthetic gene cluster is significantly downregulated at 20 h post-infection in the *G. mellonella* model, a time point at which the infection is already well established.

In conclusion, our data support a model in which OSA expression plays distinct roles at different stages of *P. aeruginosa* infection. During early infection, high expression of OSA facilitates the transition from the environment into the host and promotes evasion of the innate immune system. Once the infection is established, reduced OSA expression becomes advantageous by promoting surface attachment, biofilm formation, T3SS-mediated cytotoxicity, and antibiotic resistance, traits that collectively support persistence and the development of chronic infections. While our *ex vivo* transcriptomic data and *in vitro* expression kinetics are consistent with a temporal shift in OSA biosynthesis during infection, direct time-resolved measurements of OSA regulation *in vivo* will be needed to further substantiate this model. Mechanistically, our data suggest that cell-surface hydrophobicity may provide a common link between the observed phenotypes. All mutants with altered OSA patterns displayed significantly increased hydrophobicity (Fig. 3c), accompanied by impaired motility (Fig. 4) and reduced biofilm formation (Fig. 5). Enhanced T3SS expression offers a plausible link for the higher toxicity observed in OSA-deficient mutants (Fig. 7b), consistent with the upregulation of T3SS genes seen in late *G. mellonella* infections (Fig. 8), and with previous reports of O-antigen/T3SS crosstalk in other Gram-negative pathogens (44–46). We note, however, that these mechanistic links are based on correlative evidence from an isogenic mutant collection rather than on direct experiments. Moreover, our findings were obtained in the PA14 clade, and whether this model extends to other *P. aeruginosa* lineages and serotypes remains to be determined.

## MATERIALS AND METHODS

### Bacterial strains and culture conditions

*P. aeruginosa* PA14 deletion mutants Δ*wzz* (PA14_23360), ΔPA14_23400, Δ*orfLM* (PA14_23440–PA14_23450), Δ*orfM* (PA14_23450), Δ*orfN* (PA14_23460), Δ*wbpM* (PA14_23470), and ΔPA14_66100 were generated using a CRISPR-Cas9 assisted recombineering method developed by Pankratz et al. (15) in the UCBPP-PA14 wild-type strain background. Successful deletion was confirmed by colony PCR and Sanger sequencing. Mutants were cured from the plasmids via passaging in antibiotic-free LB medium. Plasmid loss was confirmed by testing the respective plasmid-encoded antibiotic resistances. Bacterial strains are listed in Table S7. During revision, the originally generated Δ*orfM* strain used in selected assays was found to represent an Δ*orfLM* mutant. Key LPS and motility experiments were repeated with a clean Δ*orfM* mutant, whereas assays that were not repeated are explicitly labeled as Δ*orfLM*.

### Plasmid construction and complementation of mutants

Primers are listed in Table S6 and plasmids in Table S7. DNA fragments corresponding to the *wzz* upstream region, as well as the coding regions of PA14_23400, *orfM*, and *orfN,* were generated by PCR from PA14 chromosomal DNA. Primer pairs F_wzz_SpeI/R_Pwzz_23400, F_wzz_SpeI/R_Pwzz_orfM, and F_wzz_SpeI/R_Pwzz_orfN were used to amplify the *wzz* upstream region, whereas F_Pwzz_23400/R_23400_EcoRI, F_Pwzz_orfM/R_orfM_EcoRI, and F_Pwzz_orfN/R_orfN_EcoRI were used to amplify the respective coding regions. Upstream regions and coding regions were ligated by overlapping PCR and cloned into the SpeI/EcoRI site of the mini-CTX1 vector. Plasmids for complementation of the D*wzz* and D*wbpM* mutants were generated by cloning the coding region together with the respective regulatory region (*wzz*: primer pairs F_wzz_SpeI/R_wzz_EcoRI; *wbpM*: F_wbpM_SpeI/R_wbpM_EcoRI) into the SpeI/EcoRI site of mini-CTX1.

The resulting plasmids were introduced into the respective *P. aeruginosa* mutant strains by transformation. Recombinant clones containing mini-CTX1 inserted at the *attB* locus were selected on tetracycline-containing LB plates. Excision of the unwanted plasmid sequences located between the *FRT* sites on mini-CTX1 was achieved by expressing Flp recombinase from the conjugative plasmid pFLP2. Strains that became tetracycline-sensitive had excised the plasmid backbone. Finally, loss of pFLP2 was selected on LB plates containing 5% sucrose. Colonies that were sucrose-resistant and carbenicillin-sensitive were considered cured of pFLP2.

### Swimming motility assay

Swimming motility assays were performed as previously described (10). Briefly, BM2 swim agar (0.3%) plates were left to dry at room temperature for ∼4 h prior to inoculation and incubation upright at 37°C. BM2 swim agar plates were always freshly prepared and used on the same day. After 16–18 h of incubation, an image of the plates was acquired with a transmitted-light scanner (Epson Perfection V800 Photo). The diameter of the swim zones was analyzed using ImageJ software (NIH) and averaged from six replicates per plate. The swim zone diameters were then normalized to that of the parental PA14 WT.

### Swarming motility assay

Swarming motility assays were performed as previously described (10), with minor modifications. Briefly, BM2 swarm agar (0.5%) plates were left to dry at room temperature for ∼1 h prior to inoculation and incubation upright at 30°C in a moist chamber. BM2 swarm agar plates were always freshly prepared and used on the same day. After 16 h incubation, an image of the plates was acquired with a transmitted-light scanner (Epson Perfection V800 Photo). The swarming area was analyzed using ImageJ software (NIH) and averaged from three replicates per plate. The swarming area was normalized to that of the parental PA14 WT.

### Twitching motility assay

Twitching motility assays were performed as previously described by Turnbull and Whitchurch (52), with minor modifications. Briefly, LB agar (1.5%) plates were poured the day before usage and left to dry at room temperature. The strains were stabbed through the agar layer to the bottom of the petri dish and incubated upright at 37°C for 24 h before the agar was removed and the petri dish bottom was stained with 0.1% (wt/vol) crystal violet (Sigma) to visualize the twitching area. An image of the plates was acquired with a transmitted-light scanner (Epson Perfection V800 Photo) and analyzed using ImageJ software (NIH). The twitching area was averaged from three replicates per plate and normalized to the twitching area of the parental PA14 WT.

### Single cell swimming

Single cell swimming analysis in liquid was performed as previously described (38) using light microscopy with an Axio Observer.Z1 at 40× magnification and microscopy slides from Marienfeld GmbH & Co.KG (Lauda-Königstein, Germany), which have a black epoxy resin color mask with six reaction wells. The bacterial cultures were grown to exponential phase and adjusted to an OD_600_ of 0.1, and 1 µL of the adjusted culture was spotted on the slide. Using the video function of the microscope, bacterial movement was recorded for 300 frames, which corresponds to a time of 12–16 s. For each strain, six videos were recorded, and the average mean swim velocity of the bacteria was calculated using the plugin particle tracker of the ImageJ software (NIH). The analysis of the individual particle coordinates was performed with RStudio using the Rscript myImgP.R and TrackeR7.R (53). As a negative control, PA14 Δ*fliC* was used.

### Differential gene expression analysis

Differential gene expression analysis was conducted using the R package edgeR (54) to compare gene expression profiles of different groups of clinical isolates ([21], GSE123544). The normalization of raw library sizes was achieved using counts per million (cpm) and the weighted trimmed mean of *M*-values (TMM) method (55). Genes were included in the analysis if their cpm value was higher than 1 in at least two isolates. Differential expression was determined using the edgeR function glmTreat and fold change cutoff of 1.5. *P*-values were adjusted for multiple testing using the Benjamini and Hochberg method (false discovery rate, FDR) (56). Genes with an FDR ≤ 0.05 were considered differentially expressed.

### Serum sensitivity assay

Blood from a healthy volunteer was sampled into BD Vacutainer tubes with a separation gel and clot activator and centrifuged for 10 min at 2,000 × *g* to separate cells and serum. Serum was kept at −20°C until use. As control, heat-inactivated serum (HI serum) was prepared by incubating the serum at 56°C for 2 h. Bacterial cultures were grown to exponential phase, washed with PBS, and adjusted to an initial inoculum of 2 × 10^7^ cells per 100 µL. Fifty microliters of adjusted bacterial suspension was mixed with 50 µL pre-warmed serum/HI serum and incubated at 37°C for 30 min. To stop the activity of the human complement system, the samples were put on ice for 10 min. The CFUs of the surviving bacteria were determined by plating serial dilutions.

### LPS extraction and visualization in an SDS-PAGE stained with silver stain

To identify the OSA patterns, we performed LPS extraction and SDS-PAGE followed by silver staining, as previously described (57), with some minor changes. Briefly, bacterial overnight cultures were harvested, washed, and adjusted to an OD_600_ of 2 in 1 mL PBS. The OD-adjusted bacteria were pelleted, resuspended in 150 µL dissociation buffer I (62.5 mM Tris-OH, pH 6.8, 1% glycerol, 2% SDS, 1:4 bromophenol blue [vol/vol]) containing β-mercaptoethanol, boiled in a water bath at 100°C for 10 min, and allowed to cool down to room temperature (RT) for 15 min. Ten microliters of the upper layer was carefully transferred into a new tube and 90 µL of dissociation buffer II (62.5 mM Tris-OH, pH 6.8, 1% glycerol, 1:4 bromophenol blue [vol/vol]) was added. To remove residual proteins, the extracts were treated with 2 µL proteinase K and incubated at 37°C for 1 h. Ten microliters of the extracts was then loaded onto a freshly prepared 12.5% acrylamide gel and separated by electrophoresis for approximately 2.5 h at a constant voltage of 100 V. The LPS bands were stained using the Pierce Silver Stain Kit (ThermoFisher) according to the manufacturer’s instructions. The stained gel was imaged with a transmitted-light scanner (Epson Perfection V800 Photo).

### Microbial adhesion to hydrocarbons (MATH) test

Determination of cell-surface hydrophobicity was performed using a standard MATH test as previously described (19). Briefly, bacteria were grown to exponential phase in LB, and 2 mL of the culture was harvested, washed with PBS, and adjusted to an OD_600_ of 0.5 in PBS. Two milliliters of this suspension was mixed with 400 µL of xylene and vortexed for 120 s. After 30 min at room temperature, the decrease of optical density in the aqueous phase (OD_600_) relative to that of the initial cell suspension (OD_0_) was determined, and hydrophobicity (H%) was estimated by calculating the percentage of cells adhering to xylene with the formula *H*% = [(OD_0_ − OD)/OD_0_] × 100.

### Electron microscopy

The flagella were analyzed by using scanning electron microscopy as previously described (38), with slight modifications. Bacterial cells were fixed by the addition of glutaraldehyde (final conc. 2%) for 30 min directly to the bacterial suspension and addition of formaldehyde (final conc. 5%) for another 30 min. Afterward, samples were dehydrated in a gradient series of acetone on ice (10%, 30%, 50%, 70%, and 90%), each step for 10 min, followed by two steps at room temperature with 100% acetone. Critical point drying and sputter coating with gold palladium were performed with a CPD300 from Leica and a SCD 500 from Bal-Tec. Samples were examined with a field emission scanning electron microscope Merlin from Zeiss at an acceleration voltage of 5 kV and by making use of both an Everhart Thornley HESE2 and an inlens SE detector.

For negative staining, samples were allowed to adsorb onto a thin carbon support film and washed twice with water and negatively stained with 1% (wt/vol) aqueous uranyl acetate. Samples were examined with a Libra120 Plus transmission electron microscope (Carl Zeiss, Oberkochen, Germany) at an acceleration voltage of 120 kV at calibrated magnifications.

### Confocal laser-scanning microscopy

Biofilm analysis via CLSM was performed as previously described (58) with slight modifications. Overnight cultures were adjusted to an OD_600_ of 0.01 and 100 µL of the bacterial suspensions used to inoculate a half-area, 96-well µClear microtiter plate (Greiner Bio-One). Plates were sealed with an air-permeable cover and incubated statically at 37°C in a humid atmosphere. The LIVE/DEAD BacLight Bacterial Viability Kit (Molecular Probes, Thermo Fisher) was used to stain the bacteria after 24 h incubation. Microscopy was performed shortly either after dye addition (24 h biofilms) or after further 24 h incubation (48 h biofilms). Biofilms were visualized at the bottom of the microtiter plates using the automated CLSM (SP8 System, Leica) equipped with a HC PL APO 40×/1.10 W motCORR CS2 water immersion objective. A total biofilm height of 50 µm with a z-step size of 2 µm was acquired in the center of each well using the following Ex/Em settings: Syto9 488 nm/500–550 nm and Propidium Iodide 561 nm/700–750 nm. Images were analyzed with the customized software (Developer XD 64 version 2.0.4, Definiens AG) to quantify defined parameters (including biovolume [µm^3^], area-to-volume ratio [µm^−1^], horizontal and vertical spreading [µm^2^], mean thickness [µm], biofilm roughness, and substrate coverage at the bottom of the well). Bacterial strains were tested in biological duplicates with three technical replicates each. For visualization, 3D biofilm structures were reconstructed using Imaris 9.3.1 (Bitplane/Andor). The clustering analysis was performed in R using hierarchical clustering. First, Euclidean distances between data points were calculated using the dist function. Hierarchical clustering was then applied using the “complete” linkage method via the hclust function. Finally, clusters were identified by applying a cutoff of 0.5 to the resulting dendrogram using the cutree function.

### Antibiotic susceptibility testing

Minimum inhibitory concentrations (MIC) of four antibiotics—ceftazidime, tobramycin, amikacin, and levofloxacin—were determined by microbroth dilution in 96-well plate format following the EUCAST guidelines. Epsilometer test (Etest) stripes (bioMérieux Deutschland GmbH) were used for determining MICs on LB 1.5% agar plates (wt/vol). Therefore, bacterial overnight cultures were washed with sterile PBS and adjusted to a final OD_600_ of 0.01 in PBS. One milliliter of the adjusted culture was spread on the agar. The plates were left to dry at room temperature for ∼1 h prior application of the Etest stripes to the center of the plates and incubated for 24 h at 37°C.

### Congo red binding assay

For the visualization of biofilm formation and extracellular matrix production, strains were grown on plates containing Congo red, which binds to the negatively charged complex polymers. Briefly, plates were prepared as previously described by Friedman and Kolter, with some minor modifications (59). A 1% tryptone solution without NaCl was solidified with 1% agar and supplemented with Congo red (40 µg/mL) and Coomassie Blue (20 µg/mL). Five microliters of overnight subculture was spotted in the center of the Congo red plates and incubated upside down for 10 days at 25°C. Photographs of the colonies were taken from day 3 to day 10.

### Crystal violet biofilm formation assay

Bacterial biofilm formation was quantified by crystal violet staining in a 96-well format according to a previously published method (60), with minor modifications. Briefly, bacterial overnight cultures were adjusted to an OD_600_ of 0.02 in LB, and 100 µL of the adjusted cultures was added to each well of a sterile and non-treated U-bottom PVC 96-well plate (Corning Inc., USA). Plates were sealed with an air-permeable membrane and incubated at 37°C under humid atmosphere for 24 h or 48 h, respectively. Planktonic bacteria were removed, and wells were washed twice with 200 µL sterile water prior to the addition of 150 µL of crystal violet staining solution (0.1% [wt/vol] in water). After 30 min incubation, the crystal violet solution was removed, and wells were washed twice with water and air-dried for a minimum of 2 h. For de-staining, 200 µl of 96% ethanol was added to each well and incubated for 30 min at RT. One hundred microliters of ethanol solution was transferred into a fresh 96-well flat bottom plate, and absorbance was measured at 540 nm using a microplate reader BioTek Synergy H1 (Agilent Technologies, USA). Each strain was tested in biological triplicates with four technical replicates each.

### Gentamicin protection assay

To evaluate phagocytic uptake, we used a gentamicin protection assay, as previously described (10). Briefly, RAW264.7 macrophages were cultured in DMEM (with 1 g/L glucose, 10% FCS, 2% HEPES-buffer, and 20% L929 conditioned media as a source of macrophage colony stimulation factor [M-CSF]) at a concentration of 2.5 × 10^5^ cells in a 24-well plate and allowed to grow overnight at 37°C with 7.5% CO_2_. Overnight bacterial cultures were sub-cultured and grown for 3.5 h to mid-log phase. Bacteria were washed with PBS, and cells were infected at a multiplicity of infection (MOI) of 1 (5 × 10^5^ bacteria per 100 µL in DMEM). Plates were centrifuged at 1,200 × *g* for 5 min to ensure bacteria-to-cell contact. After 1 h, 100 µL DMEM containing 300 µg/mL gentamicin was added (final concentration 50 µg/mL). Two hours post-infection, the supernatant was removed, and the macrophages were lysed with PBS containing 0.5% (vol/vol) TritonX-100. The CFUs of the intracellular bacteria were determined by plating serial dilutions.

### Lactate dehydrogenase assay

To evaluate cytotoxicity, RAW264.7 macrophages grown in phenol-red free DMEM were infected with 5 × 10^5^ bacteria at an MOI of 1 as previously described (10). After 6 h of infection, the supernatant was collected, and lactate dehydrogenase (LDH) concentration levels were measured using the CytoTox 96 Non-radioactive Cytotoxicity Assay (Promega) according to the manufacturer’s protocol. The supernatant of non-infected macrophages was used as a negative control. As a killing control, non-infected macrophages were treated with lysis buffer, and their LDH level was measured.

### *G. mellonella* infection assay

*G. mellonella* infection was performed as previously described (24). Briefly, bacteria were grown overnight, washed in sterile phosphate-buffered saline (PBS), and adjusted to a final concentration of 5,000 cells/mL in PBS. For each individual experiment, 20 larvae were infected in parallel. Twenty microliters of the *P. aeruginosa* inoculum (~100 colony-forming units) was injected into the last left proleg using a 500 µL Hamilton syringe and a 30 G needle. PBS was injected as controls. The larvae were incubated at 37°C. Death was defined by loss of movement in response to stimulation and melanization of the cuticle, which were assessed by visual inspection.

For *in vivo* treatment, *G. mellonella* larvae were infected as described above. Infections were allowed to establish for 6 h in the case of PA14 WT and 12 h for the Δ*wbpM* mutant. Subsequently, the larvae were administered in a 10 µL injection to achieve a final concentration corresponding to 10-fold MIC of either amikacin or ceftazidime, with PBS serving as a control (29). Following treatment, the larvae were incubated at 37°C. Viability was assessed by visual inspection, and bacterial loads in the hemolymph were determined.

For *ex vivo* RNA sequencing, 20 *G. mellonella* larvae were infected with PA14. After 20 h of incubation of the infected larvae at 37°C, the hemolymph was collected via puncturing the larvae. The hemolymph was pooled from 10 larvae, and 500 µL hemolymph was immediately mixed with 500 µL RNA protect (Qiagen), incubated for 10 min at room temperature, and then centrifuged for 10 min at 10,000 rpm at room temperature. The resulting pellet was stored at −80°C until used for RNA extraction.

### RNA extraction and sequencing of *ex vivo* samples

The RNeasy Mini Kit (Qiagen) and Qiashredder columns (Qiagen) were used according to the manufacturer’s instructions. Obtained RNA was quality-checked using the RNA Nano Kit on an Agilent Bioanalyzer 2100 (Agilent Technologies). To enrich the bacterial RNA, MicrobeEnrichTM Kit (ThermoFisher) was used. The bacterial ribosomal RNA was removed using the Ribo-Zero Bacteria Kit (Illumina), and cDNA libraries were generated with the ScriptSeq v2 Kit (Illumina). The samples were sequenced in single-end mode on an Illumina HiSeq 2500 device (1 × 50 bp reads). Mapping was performed using a stampy pipeline (61) with the PA14 genome as a reference.

## Data Availability

The RNA sequencing (RNA-Seq) data were uploaded to NCBI’s Gene Expression Omnibus (GSE284794).

## SUPPLEMENTAL MATERIAL

Supplemental figures mbio.01551-26-s0001.pdf Fig. S1–S9.

Supplemental tables mbio.01551-26-s0002.xlsx Tables S1–S7.

ASM does not own the copyrights to Supplemental Material that may be linked to, or accessed through, an article. The authors have granted ASM a non-exclusive, world-wide license to publish the Supplemental Material files. Please contact the corresponding author directly for reuse.
