# Supplementary material for: Role of O-specific antigen expression in Pseudomonas aeruginosa pathogenicity
Source: mBio. 2026 Oct 5;17(11):e01551-26. doi: 10.1128/mbio.01551-26 (PMC13753792; doi:10.1128/mbio.01551-26)
Supplement: Supplementary file 1 [file mbio.01551-26-s0001.pdf]

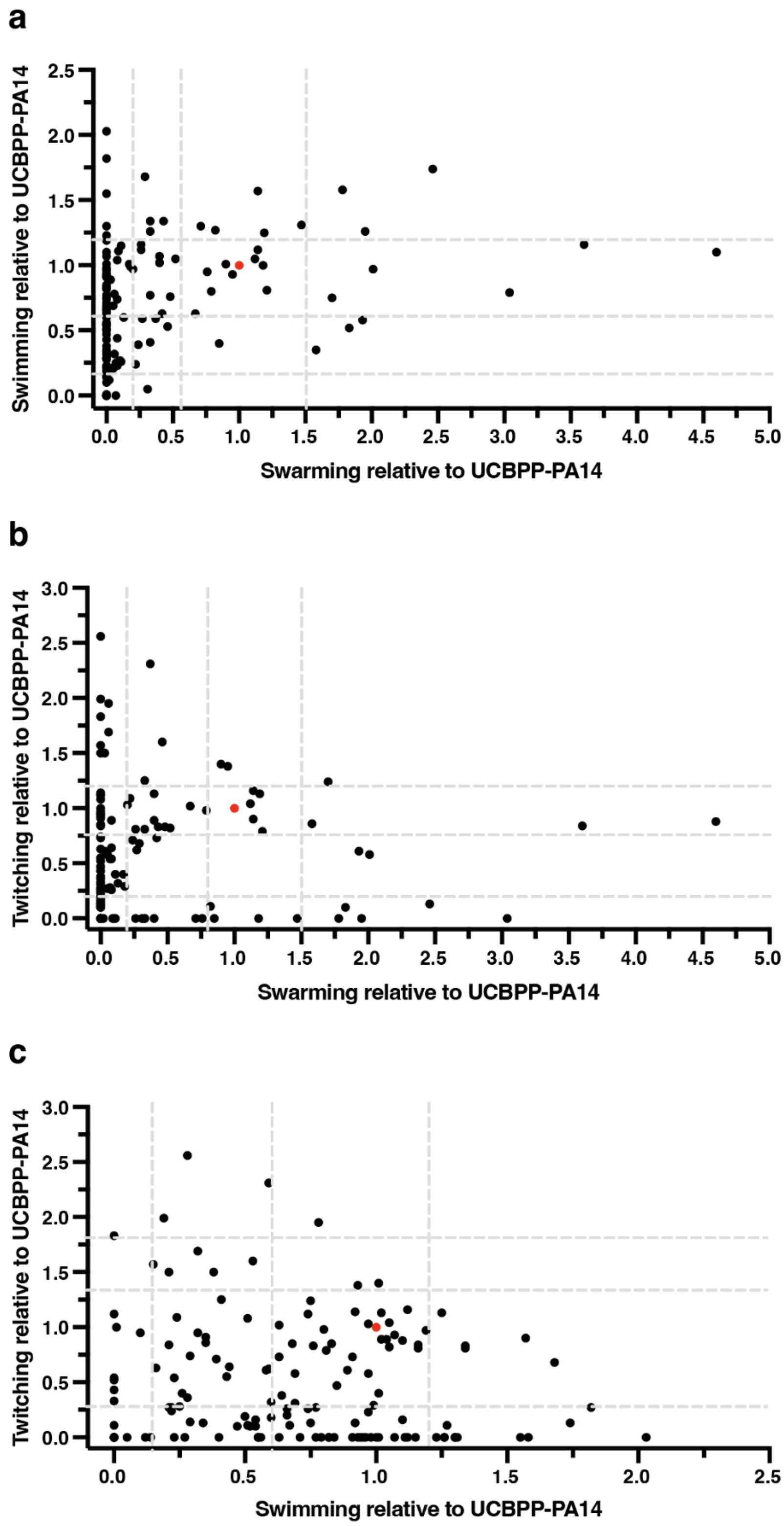

Fig. S1. Correlation analysis of the three types of motility across 144 clinical isolates. Correlation between (a) swarming and swimming motility (r = 0.405), (b) swarming and twitching motility (r = 0.197) and (c) swimming and twitching motility (r = 0.081). The reference strain PA14 is highlighted in red.

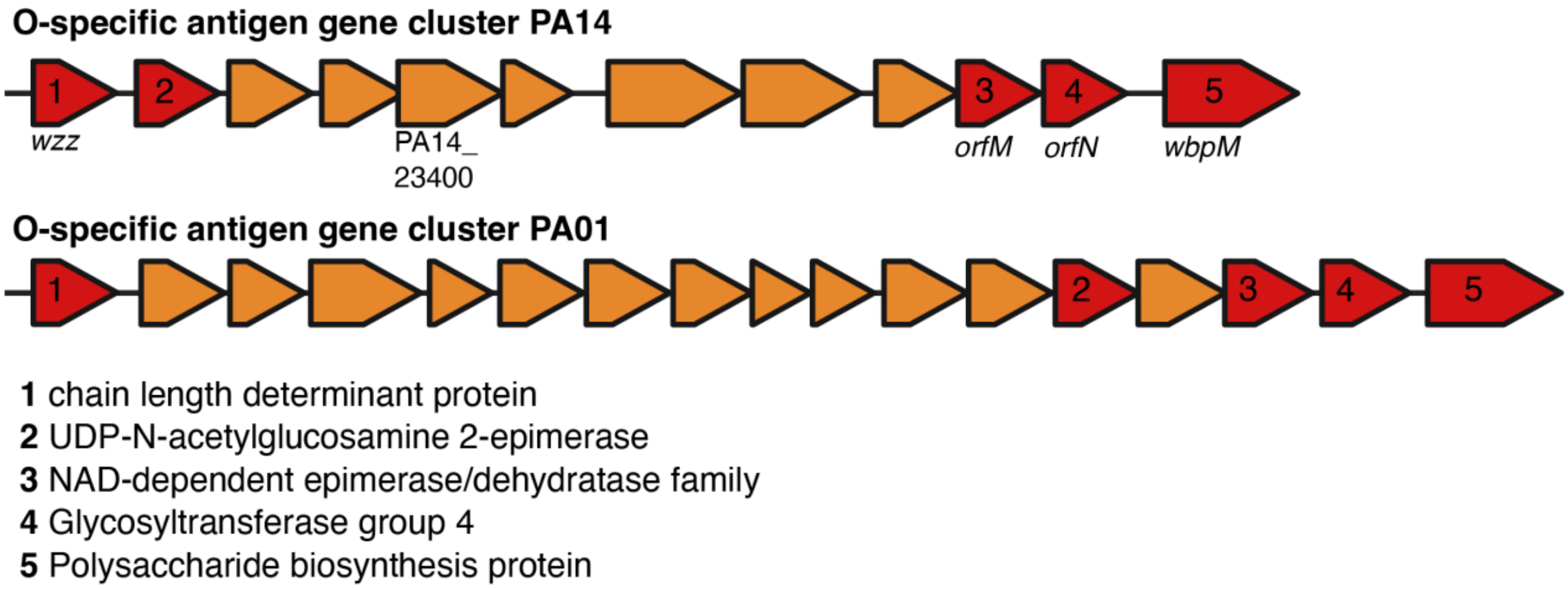

Fig. S2. Schematic overview of the O-specific antigen gene cluster in *P. aeruginosa* PA14 and PAO1, generated based on comparative genomic analysis. Conserved genes present in both type strains are shown in red; genes unique to either PAO1 or PA14 are shown in orange. The annotated functions of the conserved genes are indicated below. Previous work has shown that this genomic locus is highly variable among *P. aeruginosa* strains while being located within a conserved chromosomal region (Raymond *et al.*, 2002; PMID 12057956).

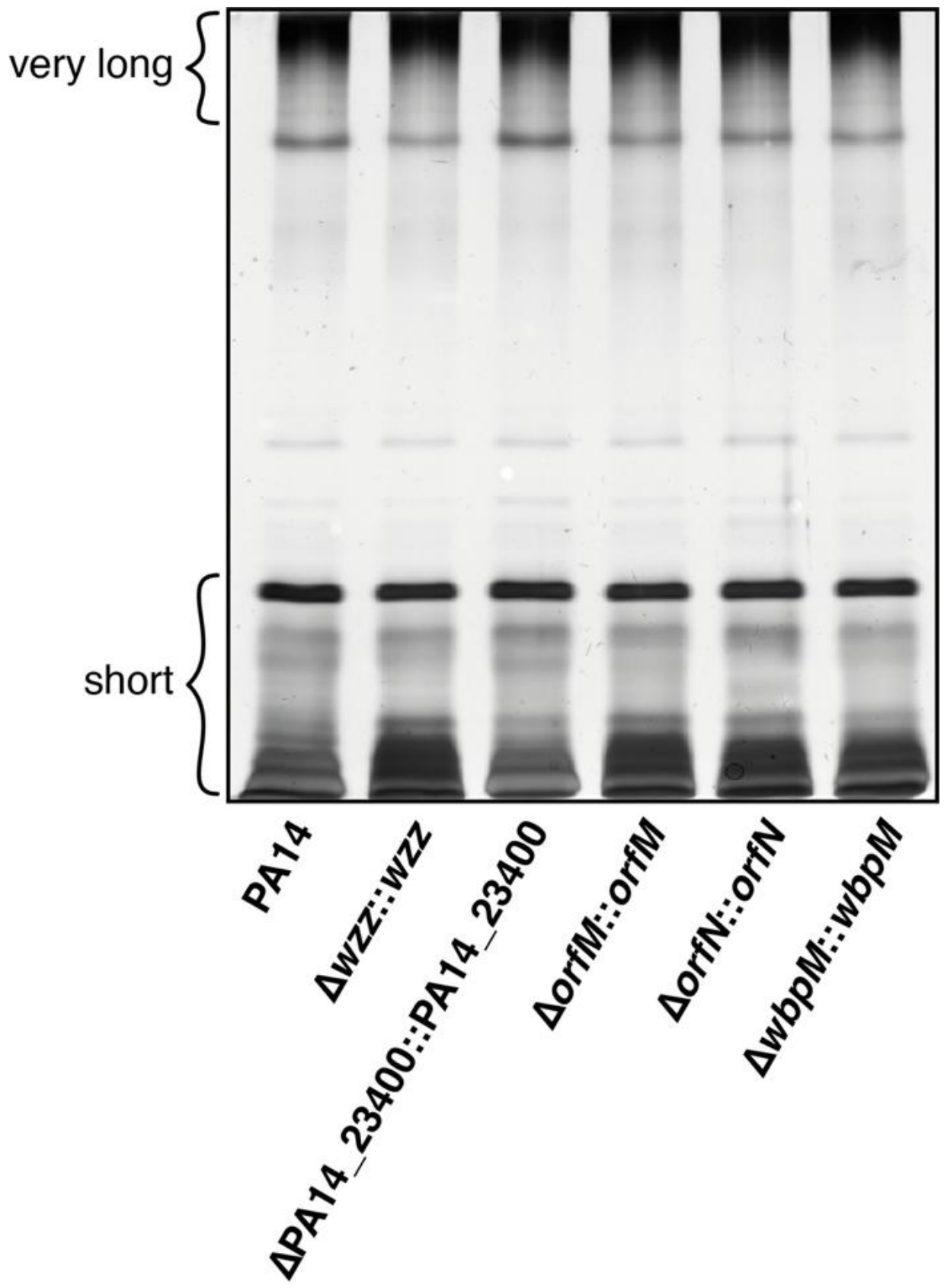

Fig. S3. Complementation restores the O-specific antigen pattern of OSA biosynthesis mutants. Silver-stained 12.5% SDS-PAGE used to characterize short and very long chains of the O-specific antigen in PA14 and the indicated chromosomally complemented mutants. Complementation of Δ*wzz*, ΔPA14_23400, Δ*orfM*, Δ*orfN*, and Δ*wbpM* restored the WT-like OSA pattern. The positions of short and very long OSA chains are indicated.

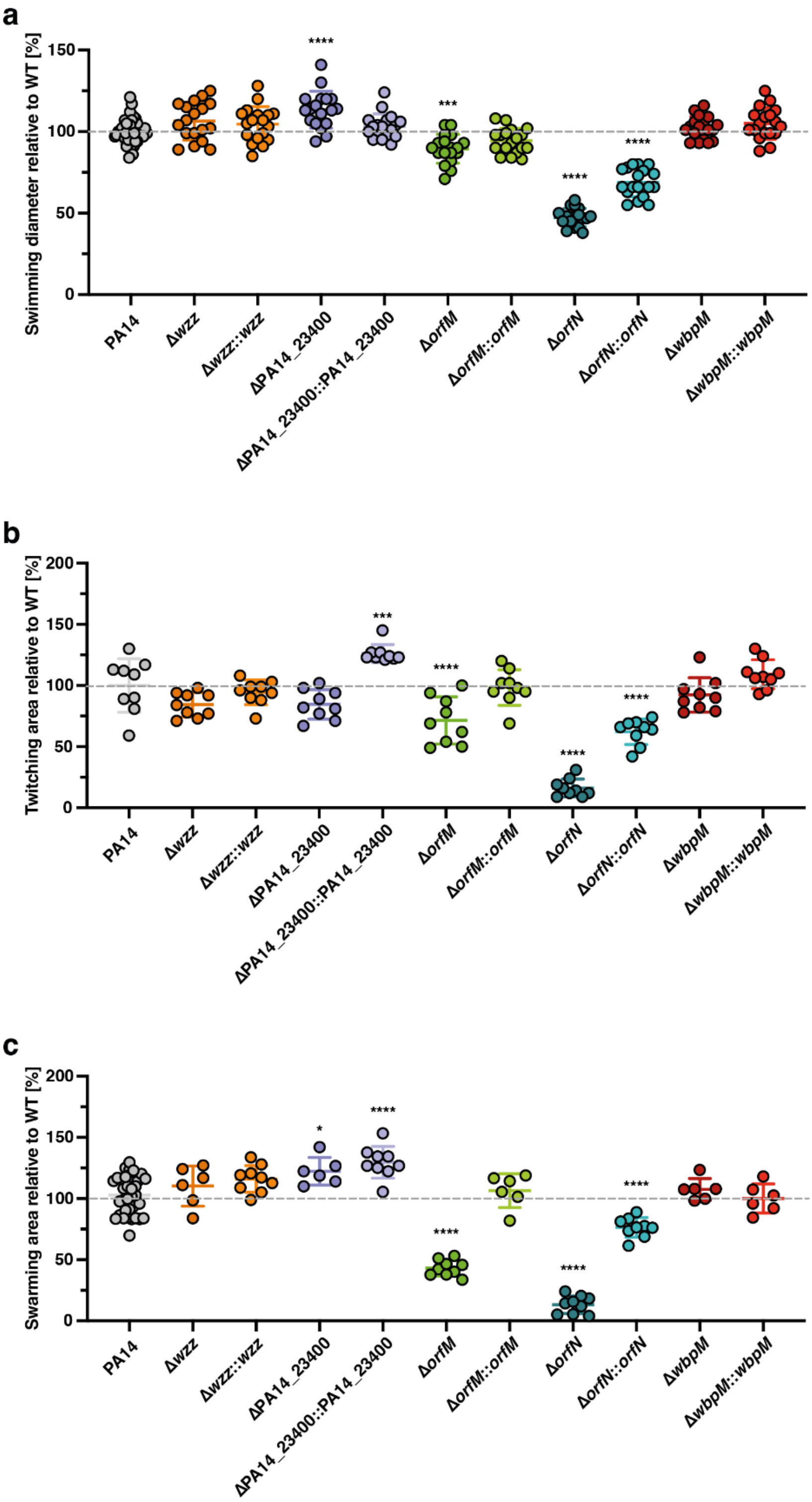

Fig. S4. Complementation restores motility phenotypes of O-specific antigen biosynthesis mutants. (a) Swimming motility of PA14, the indicated O-specific antigen biosynthesis mutants, and their chromosomally complemented strains relative to the WT after 18 h of incubation on 0.3% swimming agar plates at 37 °C. (b) Twitching motility relative to the WT after 48 h of incubation on 1.5% twitching agar plates at 37 °C. (c) Swarming motility relative to the WT after 16 h of incubation on 0.5% swarming agar plates at 30 °C under humid atmosphere. Dashed lines indicate the WT level. * $p<0.05$, *** $p<0.001$, **** $p<0.0001$, one-way analysis of variance (ANOVA) (post-hoc test: Dunnett).

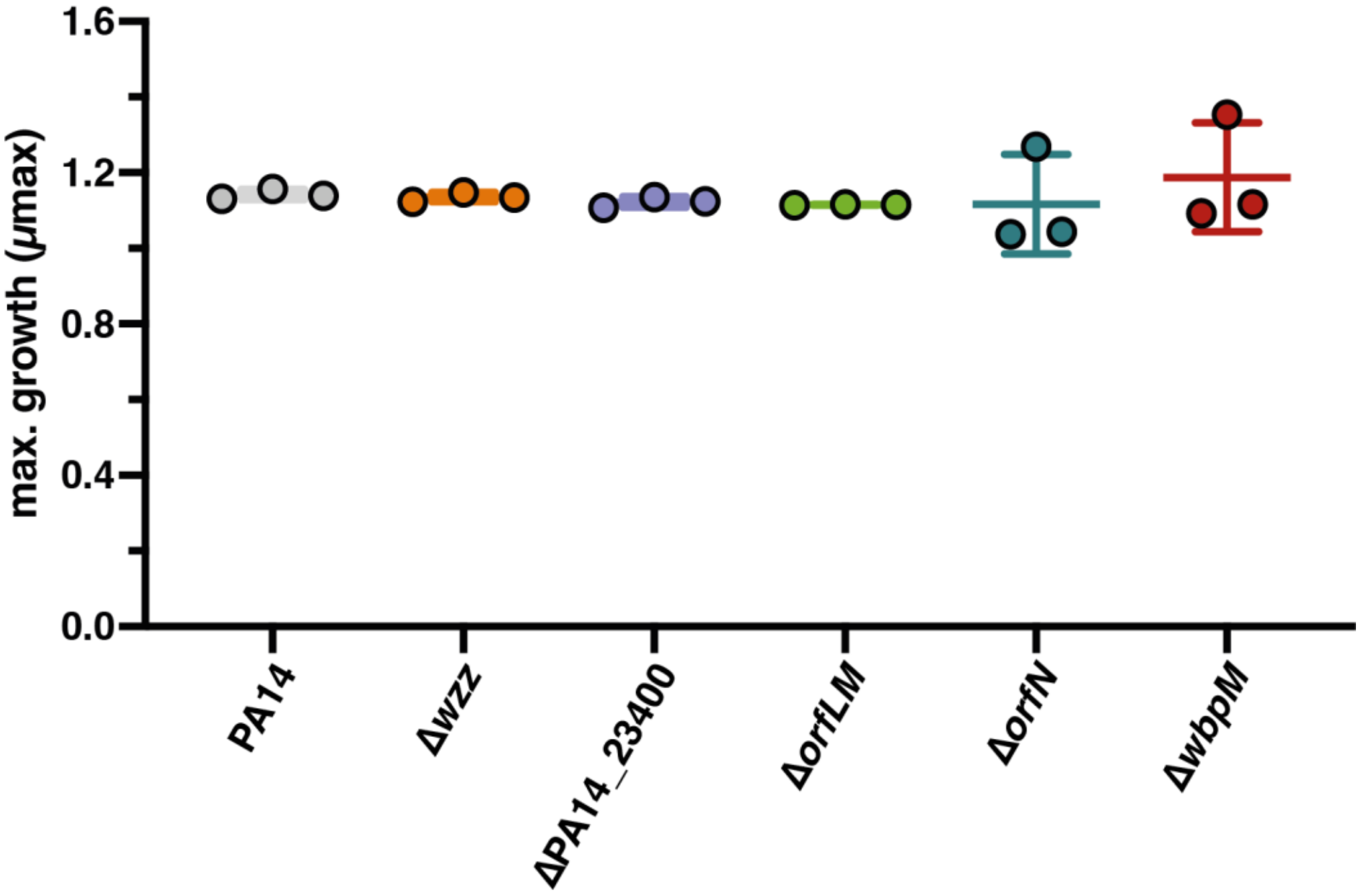

Fig. S5. Growth of the O-specific antigen deletion mutants. Maximum growth rate (µmax) of PA14, *Δwzz,* ΔPA14_23400, Δ*orfLM*, Δ*orfN* and Δ*wbpM* in LB medium. Data represent three biological replicates per strain.

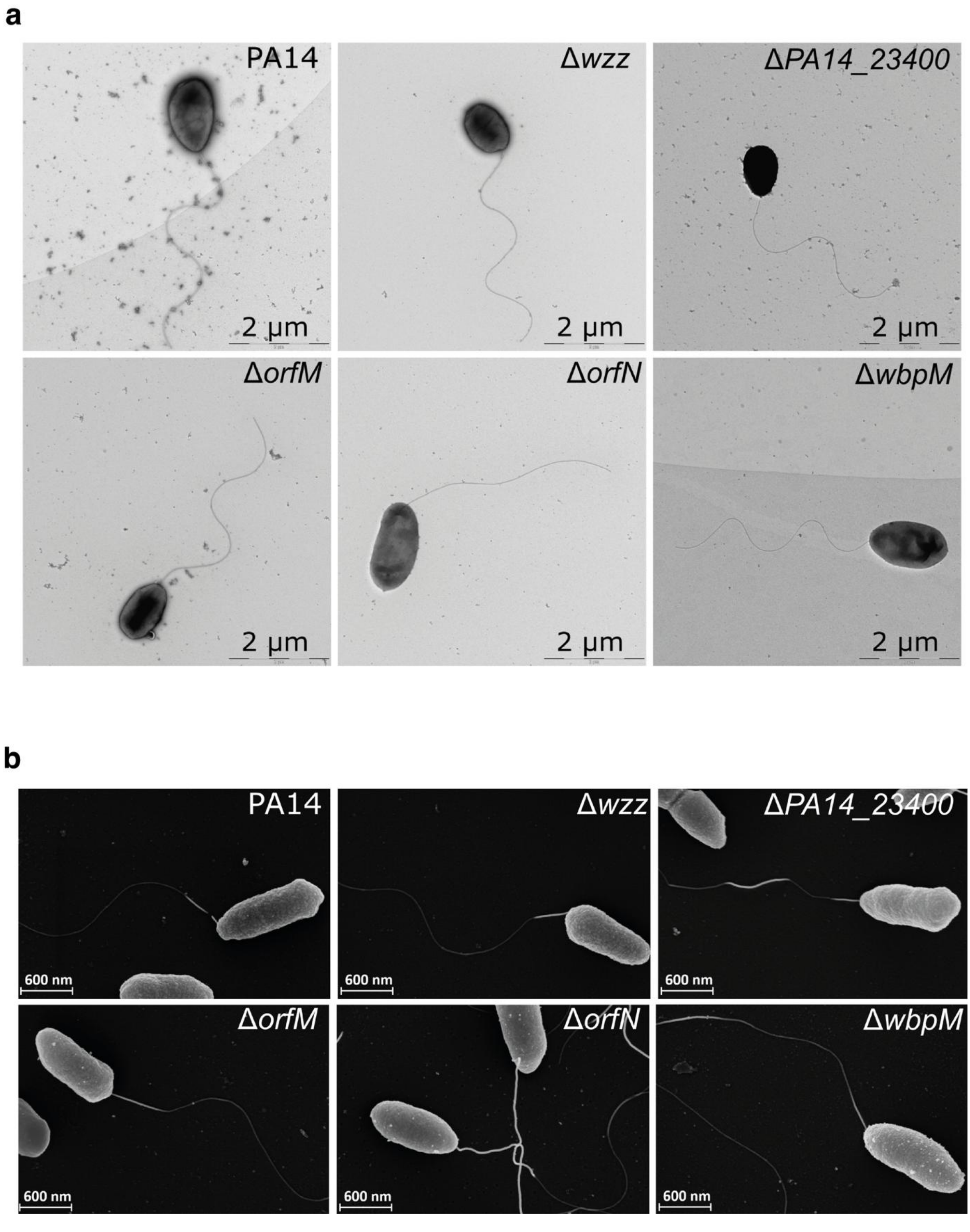

Fig. S6. Representative electron microscopy pictures of the flagellum of the O-specific antigen mutants. Flagella were visualized using (a) negative-staining electron microscopy and (b) scanning electron microscopy. Settings: Mag = 32.00 KX, EHT = 5.00 kV and WD = 3.3 mm.

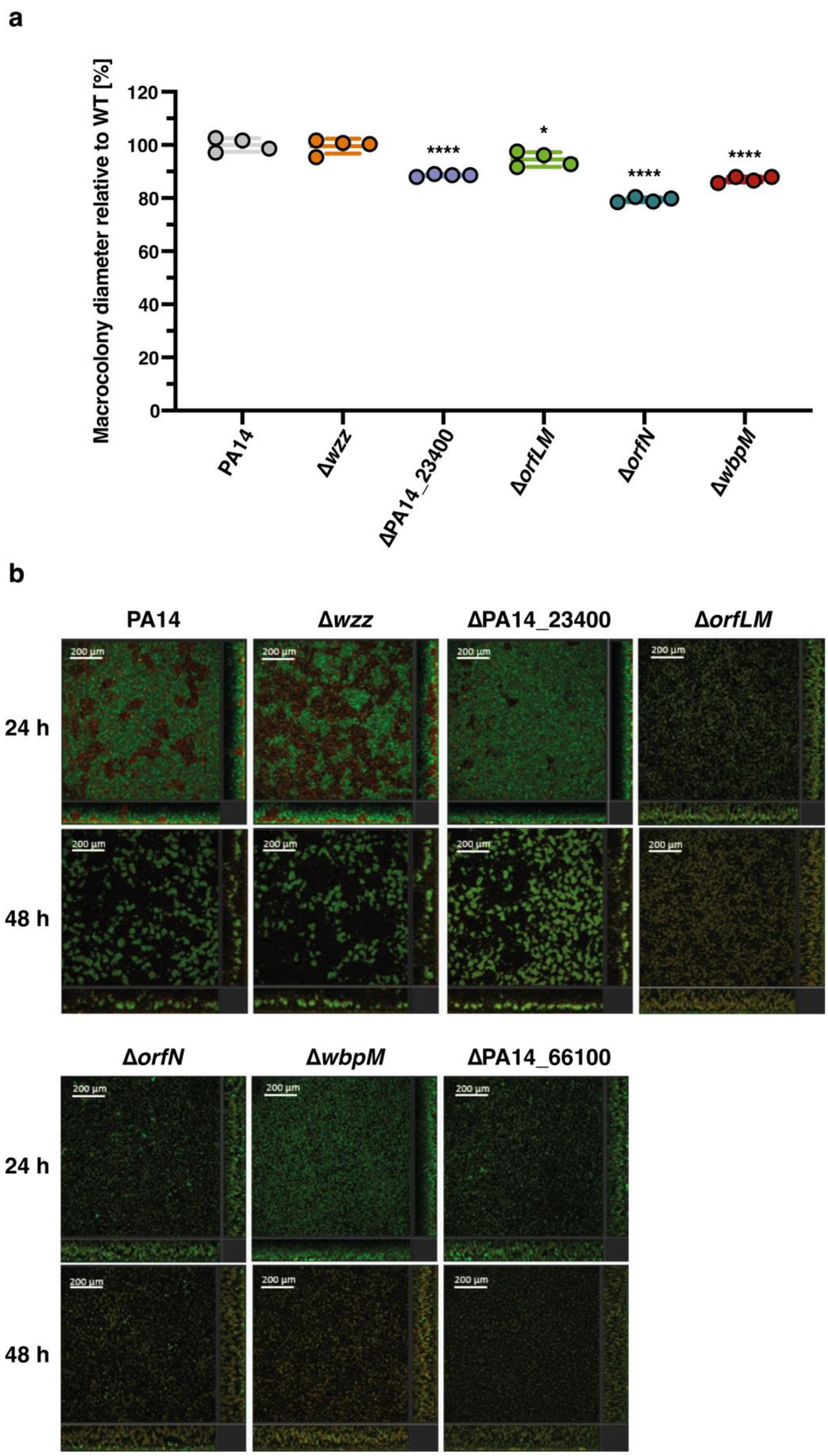

Fig. S7. Altered O-specific antigen biosynthesis affects macrocolony size and biofilm architecture**.** (a) Diameter of colony biofilm macrocolonies after 10 days of incubation on Congo red agar plates. (b) Representative CLSM images of biofilms formed after 24 and 48 h in microtiter plates, acquired following live/dead staining. Live cells appear in green (Syto9) and dead cells appear in red (propidium iodide: PI). 3D reconstructions were generated using Imaris software. ** p<0.01, **** p<0.0001, one-way analysis of variance (ANOVA) (post-hoc test: Dunnett).

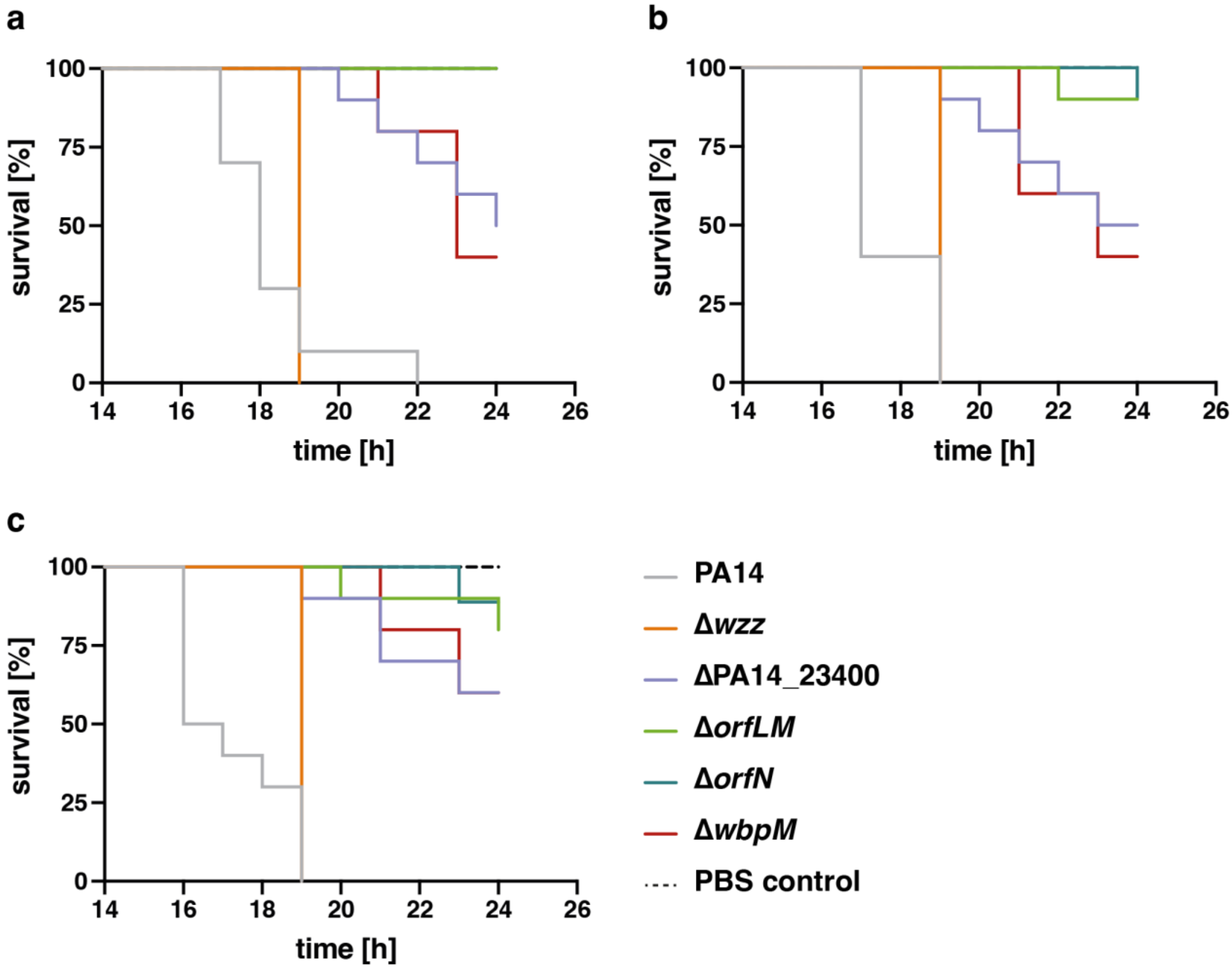

Fig. S8. Lack of O-specific antigens results in reduced virulence in the *G. mellonella* infection model. Kaplan-Meier survival curves of *G. mellonella* larvae injected with PA14 WT or O-specific antigen mutants at an MOI of 100. Time-dependent survival over 24 h is shown for three independent experiments (A-C). Each curve represents n = 10 larvae per experiment.

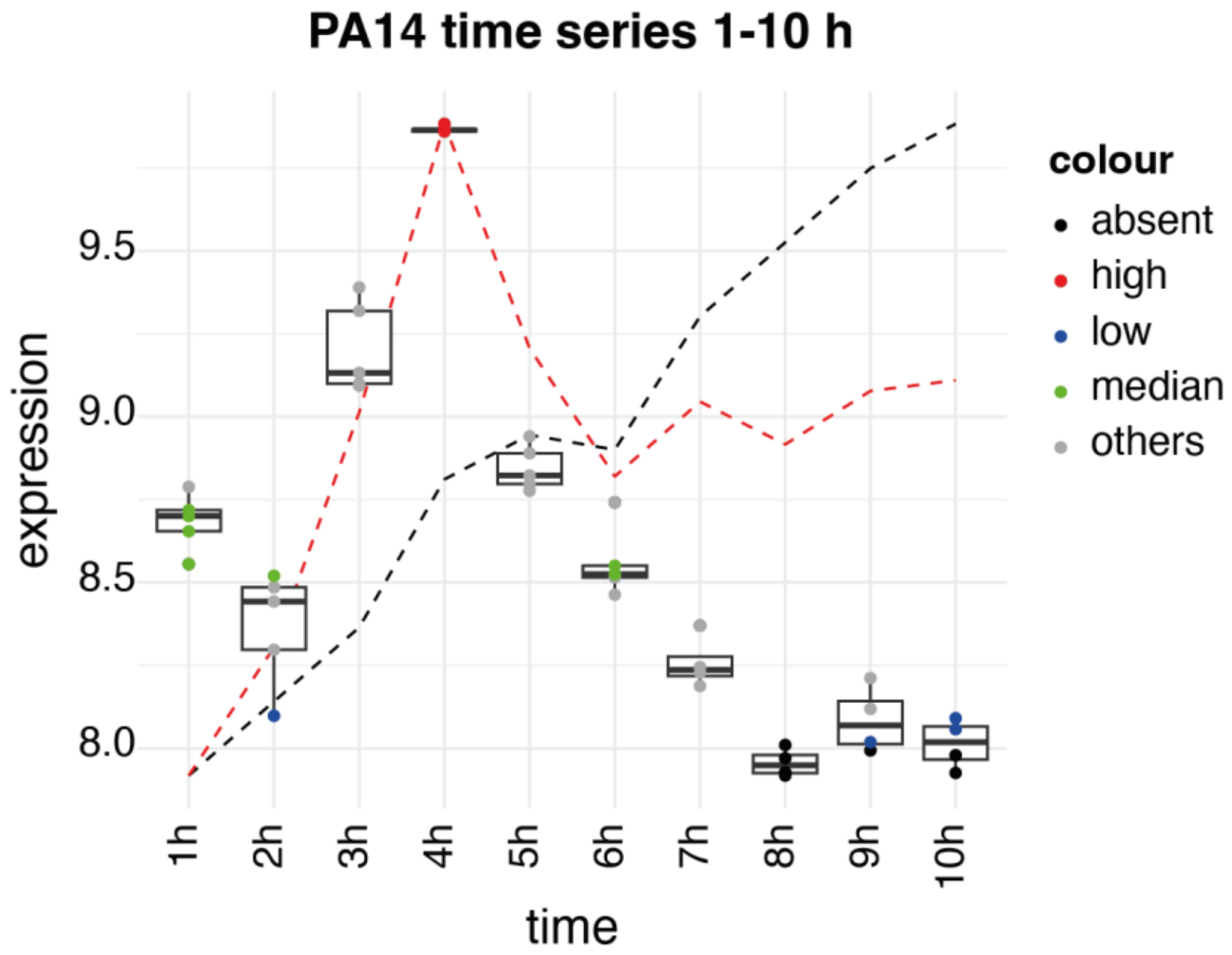

Fig. S9. Representative expression analysis of *wbpM* over a 10-hour time series. PA14 WT was cultured in LB medium, and samples were collected hourly for OD_{600} measurement, CFU determination, and RNA sequencing. The y-axis displays absolute *wbpM* expression as log2 values derived from the RNA-seq data; the x-axis represents time (hours). The black dashed line indicates the relative OD_{600}, and the red dashed line represents the relative CFUs.
